# Investigation on Quantitative Structure Activity Relationships and Pharmacophore Modeling of a Series of mGluR2 Antagonists

**DOI:** 10.3390/ijms12095999

**Published:** 2011-09-16

**Authors:** Meng-Qi Zhang, Xiao-Le Zhang, Yan Li, Wen-Jia Fan, Yong-Hua Wang, Ming Hao, Shu-Wei Zhang, Chun-Zhi Ai

**Affiliations:** 1 Department of Materials Science and Chemical Engineering, Dalian University of Technology, Dalian, Liaoning 116024, China; E-Mails: manna1989@mail.dlut.edu.cn (M.-Q.Z.); rosemarryfan@gmail.com (W.-J.F.); dluthm@yeah.net (M.H.); zswei@dlut.edu.cn (S.-W.Z.); 2 Department of Mathematical Sciences, Dalian University of Technology, Dalian, Liaoning 116024, China; E-Mail: xlfree@foxmail.com; 3 Lab of Pharmaceutical Resource Discovery, Dalian Institute of Chemical Physics, Graduate School of the Chinese Academy of Sciences, Dalian, Liaoning 116023, China; E-Mail: aicy@dicp.ac.cn; 4 Center of Bioinformatics, Northwest A&F University, Yangling, Shaanxi 712100, China; E-Mail: yhwang@dlfu.edu.cn

**Keywords:** 3D-QSAR, mGluR2 antagonist, CoMFA, CoMSIA, pharmacophore modeling

## Abstract

MGluR2 is G protein-coupled receptor that is targeted for diseases like anxiety, depression, Parkinson’s disease and schizophrenia. Herein, we report the three-dimensional quantitative structure–activity relationship (3D-QSAR) studies of a series of 1,3-dihydrobenzo[ b][1,4]diazepin-2-one derivatives as mGluR2 antagonists. Two series of models using two different activities of the antagonists against rat mGluR2, which has been shown to be very similar to the human mGluR2, (activity I: inhibition of [^3^H]-LY354740; activity II: mGluR2 (1*S*,3*R*)-ACPD inhibition of forskolin stimulated cAMP.) were derived from datasets composed of 137 and 69 molecules respectively. For activity I study, the best predictive model obtained from CoMFA analysis yielded a *Q*^2^ of 0.513, *R*^2^ _ncv_ of 0.868, *R*^2^ _pred_ = 0.876, while the CoMSIA model yielded a *Q*^2^ of 0.450, *R*^2^ _ncv_ = 0.899, *R*^2^ _pred_ = 0.735. For activity II study, CoMFA model yielded statistics of *Q*^2^ = 0.5, *R*^2^ _ncv_ = 0.715, *R*^2^ _pred_ = 0.723. These results prove the high predictability of the models. Furthermore, a combined analysis between the CoMFA, CoMSIA contour maps shows that: (1) Bulky substituents in R_7_, R_3_ and position A benefit activity I of the antagonists, but decrease it when projected in R_8_ and position B; (2) Hydrophilic groups at position A and B increase both antagonistic activity I and II; (3) Electrostatic field plays an essential rule in the variance of activity II. In search for more potent mGluR2 antagonists, two pharmacophore models were developed separately for the two activities. The first model reveals six pharmacophoric features, namely an aromatic center, two hydrophobic centers, an H-donor atom, an H-acceptor atom and an H-donor site. The second model shares all features of the first one and has an additional acceptor site, a positive N and an aromatic center. These models can be used as guidance for the development of new mGluR2 antagonists of high activity and selectivity. This work is the first report on 3D-QSAR modeling of these mGluR2 antagonists. All the conclusions may lead to a better understanding of the mechanism of antagonism and be helpful in the design of new potent mGluR2 antagonists.

## 1. Introduction

Glutamate is a useful excitatory neurotransmitter of the nervous system, although its excessive amount in the brain can lead to cell death through a process called excitotoxicity, which consists of the over stimulation of glutamate receptors. Excitotoxicity occurs in neurological diseases such as Alzheimer’s disease, Parkinson’s disease and multiple sclerosis [1]. The major excitatory neurotransmitter substance in the mammalian central nervous system is L-glutamate, which acts on receptors that are highly heterogeneous and of two types: ionotropic glutamate receptors (iGluRs) that mediate fast-synaptic transmission in most neuronal synapses and metabotropic glutamate receptors (mGluRs) which are G-protein Coupled Receptors linked to multiple second messengers and modulate the ion channel currents [2,3]. Until now, at least eight mGluRs have been described, subdivided into three groups based on their primary structure, second-messenger coupling, and pharmacology. Group I receptors include mGluR 1 and 5, group II mGluR2 and 3, and group III mGluR 4, 6, 7 and 8 [4]. These mGluRs play essential neuromodulatory roles throughout the brain, as such they are attractive targets for therapeutic intervention for a number of psychiatric and neurological disorders including anxiety, depression, Fragile X, Syndrome, Parkinson’s disease and schizophrenia [5].

In the latest decades, pharmacological agents acting at specific mGluR subtypes have been developed. In particular, mGluR2/3 agonists showing antipsychotic properties and mGluR2/3 antagonists may be useful as antidepressants and cognitive enhancers as demonstrated in different animal models [6]. These agents include the group II -selective agonist LY354740 and antagonist LY341495 [7]. These agonists have been reported to have neuroprotective, anxiolytic/anti-panic and anti-Parkinsonism properties, as well as anti-psychotic potential [8–10]. However, recently studies showed that LY354740 has been demonstrated to impair the spatial navigation memory using the water maze and produces a dose-dependent impairment of working memory in the delayed match to position (DMTP) task in rats, although in humans there are no reports to date that mGluR2/3 agonists impair the cognitions [6,11].

Recently, acting as mGluR2 and 3 non-competitive antagonists, a series of in vivo active and well tolerated 1,3-dihydro-benzo[b][1,4]diazepin-2-one derivatives were synthesized by Woltering T.J. *et al* [12]. They were reported to partially inhibit the binding of the selective agonist [^3^H]-LY354740 to rat mGluR2, fully block the effect of LY354740, (1*S*,3*R*)-ACPD and L-glutamate in cAMP assays. These mGluR2/3 antagonists show pharmacological potentiality by blockade of the mGluR2/3 agonist LY354740-induced hypoactivity and improvement of a working memory deficit induced either by LY354740 or scopolamine in the delayed match to position task (DMTP). Also, combination studies of the antagonists with a cholinesterase inhibitor shows apparent synergistic effects on working memory impairment induced by scopolamine. In addition, among the series of mGluR2 antagonists reported, compound 8am was found to exhibit mild antidepressant-like activity in the mouse, indicating further potential use of mGluR2/3 antagonists as antidepressant drugs [12].

Moreover, mGluR2 antagonists have been considered as major elements of a pharmaceutical composition to treat and prevent acute or chronic neurological disorders including Alzheimer’s disease and mild cognitive impairment (United States Patent 7235547). Also, Addex Pharmaceuticals (a pharmaceutical cooperation) has made plans to move mGluR2 antagonist into clinical trials for Alzheimer’s disease [13]. The great potential of further biological and pharmaceutical function of mGluR2 antagonist is still under exploration. Therefore, the antagonism of the series of 1,3-dihydrobenzo[ b][1,4]diazepin-2-one derivatives has great value to investigate.

Quantitative structure activity relationship (QSAR) has been widely used to find out various interactive fields making impacts on activity, to predict the activities of the inhibitors and thus to help forecasting and designing of better specific ligands [14–16]. It is a mathematical model of correlation, statistically validated, between the variation on chemical structure and the activity profile of a series of compounds [17]. Nowadays, three-dimensional (3D) quantitative structure activity relationship (3D-QSAR) techniques, especially comparative molecular field analysis (CoMFA) and comparative molecular similarity analysis (CoMSIA) are routinely used in modern drug design [18]. Furthermore, pharmacophore model is another method to investigate into the structure-activity relationship of molecules. Common pharmacophoric features can be obtained from pharmacophore model based on known active compounds, providing guidance for the rational design of novel selective chemicals. In addition, molecular docking, which is also attempted in this paper, is utilized more and more in current drug design process. Here we focused on the study of a series of 8-ethynyl-1,3-dihydrobenzo[ b][1,4]diazepin-2-one derivatives that has been reported as new potent non-competitive mGluR2/3 antagonist [12]. The aim of the present study is to use the 137 newly fused compounds mentioned above as a data set to identify their requisite structural features affecting the mGluR2 antagonist effects by a combination of several in silico approaches including CoMFA, CoMSIA and pharmacophore modeling. Structure-activity relationship concerning mGluR3 is not investigated, for there are no reported experimental values of activity presently. As far as we know, this study provides the first 3D-QSAR study and pharmacophore modeling for the series of new mGluR2 antagonists.

## 2. Results and Discussion

To judge whether a QSAR model is highly qualified, several statistical parameters including especially the cross-validated correlation coefficient (*Q*^2^), non cross-validated correlation coefficient (*R*^2^ _ncv_), standard error of estimate (SEE) and F-statistic values as well as the optimum number of components (OPN) should be evaluated. Various 3D-QSAR models were generated and the best model was selected based on the statistically significant parameters obtained. For both 3D-QSAR studies, good correlations were observed in the obtained CoMFA and CoMSIA models demonstrated by the high values of *Q*^2^, *R*_ncv_, *R*_pred_ and other statistical results. Table 1 summarizes the statistical results of the CoMFA and CoMSIA analyses.

### 2.1. Results for Activity I

#### 2.1.1. 3D-QSAR Statistical Results

During the molecular modeling process, 110 compounds out of the total 137 mGluR2 antagonists were used as training set and the remaining 27 compounds were used as test set (shown in Tables S1–S4 in supporting information). The best results were obtained at a column filtering of 1 kcal/mol for both steric and electrostatic fields for CoMFA analysis. PLS analysis showed a *Q*^2^ value of 0.513 with 7 components for CoMFA, indicating a good internal predictive capacity of the model. A high correlation coefficient of 0.868 for the non-cross-validated model shows its self-consistency.

In addition, other statistical results including a SEE of 0.296 and an *F*-test value of 96.022 are reported (Table 1). Steric field descriptors explain 0.488 of the variance, while the electrostatic descriptors contribute 0.461, suggesting a balanced percentage of the influence exerted by the two fields on the activity of the antagonist. In addition, ClogP contributes a minor percentage of 5.1, indicating that the hydrophobicity of compound does affect its antagonistic activity to some extent.

For the CoMSIA analysis, all combinations of the five different field descriptors (the steric, electrostatic, hydrophobic, H-bond-donor and H-bond acceptor) were used, with attempt to seek for the best CoMSIA model while avoiding the risk of possible omitting of some optimal ones. As a result, out of all CoMSIA models established using the same training set as used in the CoMFA analysis, the optimal CoMSIA model was made use of all five field parameters, and reveals a validated *Q*^2^ of 0.450 for 8 components, a *R*_ncv_ ^2^ of 0.899, F value of 101.353 and SEE of 0.273. The electrostatic field was demonstrated contributing a lot to the model with a sum of 27.7 percentages. In addition, the hydrogen bond acceptor field descriptor fulfills its role by correlating with the mGluR2 antagonist activity with a fraction of 0.256. Steric field contributes 11.2 percent of the variances. H-bond donor field and acceptor field contribute 0.184 and 0.135 respectively.

**Figure 1:**
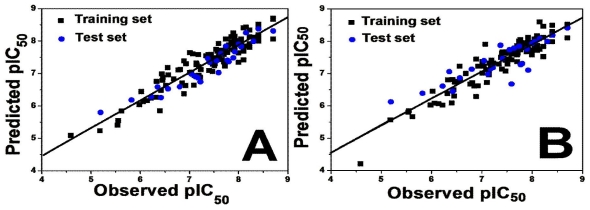

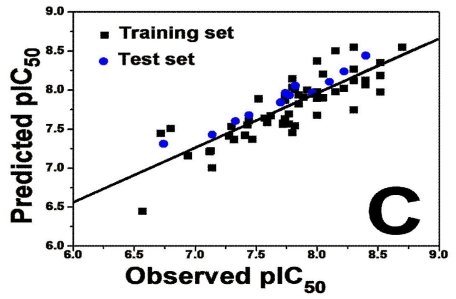
The ligand-based correlation plots of the predicted versus the actual pIC_50_ values using the training (filled black square) and the test (filled blue circle) set compounds based on (**A**) CoMFA for activity I (Dataset A: 137 compounds); (**B**) CoMSIA models for activity I (Dataset A: 137 compounds); (**C**) CoMFA for activity II (Dataset B: 69 compounds).

All the statistical parameters of CoMFA and CoMSIA obtained show that the models generated are reasonable. Furthermore, the accuracy of these models was elucidated using an external test set of 27 compounds. With a high predictive coefficient *R*^2^ _pred_ of 0.876 (*R*^2^ _ncv_ = 0.868) for CoMFA and 0.735 (*R*^2^ _ncv_ = 0.899) for CoMSIA achieved, the test sets potently validate the efficacy of the CoMFA and CoMSIA models. Figure 1A, B illustrate the correlation plots of the experimental versus the predicted pIC_50_ values of the training (filled black square) and test sets (filled blue circle) for the optimal CoMFA and CoMSIA models, respectively. Clearly, a good correlation was observed from this figure since the predicted values are almost as accurate as the experimental activities for the whole dataset, and all points are rather uniformly distributed around the regression line. This good agreement between the predicted and experimental activity data proves the satisfactory predictive ability of both the CoMFA and CoMSIA models.

#### 2.1.2. Contour Maps for Activity I

**Figure 2:**
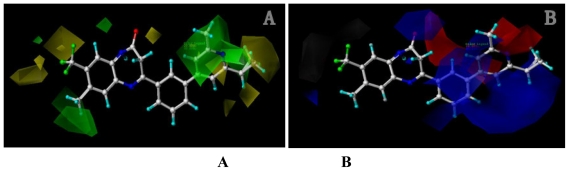
CoMFA StDev*Coeff contour maps for activity I. (**A**) Steric (green/yellow) contour map in combination with compound **8ao**. Green contours indicate regions where bulky groups increase activity; yellow contours indicate regions where bulky groups decrease activity; (**B**) Electrostatic contour map (red/blue) in combination with compound 8ao. Red contours indicate regions where negative charged groups increase activity; blue contours indicate regions where positive charged groups increase activity.

**Figure 3:**
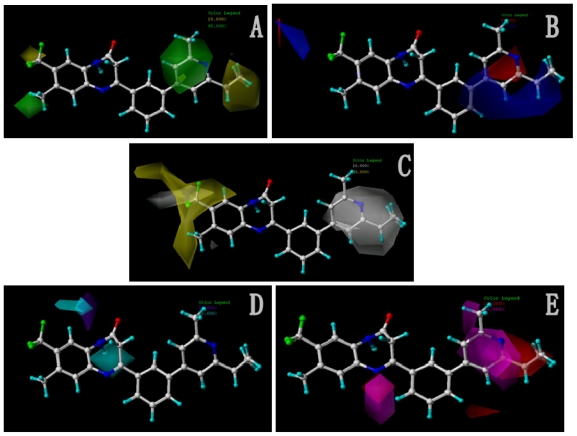
CoMSIA StDev*Coeff contour maps for activity I, with **8ao** as reference molecule. (**A**) Steric (green/yellow) contour map. Green contours indicate regions where bulky groups increase activity; yellow contours indicate regions where bulky groups decrease activity; (**B**) Electrostatic contour map. Red contours indicate regions where negative charges increase activity; blue contours indicate regions where positive charges increase activity; (**C**) Hydrophobic contour map. Yellow contours indicate regions where hydrophobic substituents enhance activity; white contours indicate regions where hydrophilic substituents enhance activity; (**D**) Contour maps illustrating H-bond donor features. The cyan contour represents the H-bond donor favored region, purple indicates the disfavored region; (**E**) CoMSIA contour maps illustrating H-bond acceptor features. The magenta contour indicates regions where H-bond acceptor groups increase activity, the red contour indicates the disfavored region for H-bond acceptor groups.

Contour maps were generated as scalar products of coefficients and standard deviation, associated with each CoMFA or CoMSIA column. The maps generated depict regions having scaled coefficients 80% (favored) or 20% (disfavored). To aid in visualization, the most active antagonist in the series (compound **8ao**) is shown superimposed with the CoMFA (Figure 2) and CoMSIA contour maps (Figure 3). The coefficient contour plots are essential to identify the important regions where some changes in the steric, electrostatic and hydrophobic fields may affect the biological activity. This is particularly important when increasing or reducing the activity of a compound by changing its molecular structural features contributing to the interaction between the ligand and the active site region of a receptor.

##### 2.1.2.1. CoMFA Contour Maps

The contour maps of CoMFA denote the region in the space where the aligned molecules would favorably or unfavorably interact with the receptor. Contribution for favorable and unfavorable interactions with the receptor in terms of steric (80% green, 20% yellow) and electrostatic (80% blue and 20% red) were shown.

For steric contour map, the green contour mapped near R_7_ substituent suggests that a sterically bulky group is favorable and increases the activity. This is well illustrated by the example that compound **8at** (pIC_50_ = 8.40, *R*_7_ = −OEt) has higher activity than any other compounds with smaller substituents at the position R_7_, including especially the **8as** (pIC_50_ = 8.00), which has the same structure with **8at** except for absence of groups in position R_7_. In addition, the large green region near the heterocyclic ring (ring D) and position A of the ring suggests that the activity I also benefits from bulky groups of these sites. This can be demonstrated by that fact that compounds of groups 7, 8 with a heterocyclic ring D at R_3_ position generally have higher activities than group 14 (with no ring at R_3_ position). Also, in case where a heterocyclic ring D is commonly shared, **8am**, **8an**, **8ao** (pIC_50_ = 8.70, position A = −CH_3_) respectively bear higher activities than **8i** (pIC_50_ = 8.40), **8j** (pIC_50_ = 8.10), **8m** (pIC_50_ = 8.00) with no groups at position A. A yellow contour map shown towards the position R_8_ indicates that the longer chain substituents towards this spatial distribution decrease the activity. Compounds **7g** (pIC_50_ = 6.68) and **7r** (pIC_50_ = 6.33) showing less antagonist activity are just due to their bulky propyl substituents that are projected in the yellow region. This is also the same with the yellow blocks appearing at position B of ring D. A drop in activity of compound **8ax** (pIC_50_ = 8.22, position B = −CH_3_) when compared with **8at** (pIC_50_ = 8.40, position B = −H) can illustrate this point.

The CoMFA electrostatic contour plots for highly active compound **8ao** are displayed in (Figure 2B). The large blue polyhedron partially encompassing the pyridine ring D indicates a favor for electropositive substituents in this region. Compounds **8ap** (pIC_50_ = 7.85), **8t** (pIC_50_ = 7.40), **8u** (pIC_50_ = 6.56) are less potent than compounds **8am**, **8an**, **8ao** (pIC_50_ = 8.70) just due to their strong negative groups (−OH and −NMe_2_) in these areas. Red polyhedron around the N atom at position 4 of ring D indicates that substituents should be electron deficient for high binding affinity with protein, which can be illustrated by the fact that **8h** (pIC_50_ = 8.40, with N atom at position 4 of ring D) and **8ae** (pIC_50_ = 8.30, with −OMe) enjoy higher activities than **8y** (pIC_50_ = 7.59, with C atom at position 4) and **8aa** (pIC_50_ = 7.89, with −Me) respectively.

##### 2.1.2.2. CoMSIA Contour Maps

CoMSIA contribution maps denote those areas within the specified region where the presence of a group with a particular physico-chemical property will be favored or disfavored for good biological activity. With **8ao** as reference in the background, CoMSIA steric (Figure 3A) and electrostatic contour maps (Figure 3B) show favorable and unfavorable regions that are highly similar to maps derived from the CoMFA analysis above, thus are not discussed here.

Figure 3C shows the CoMSIA hydrophobic field contour map, where the yellow (hydrophobic favorable) and white (hydrophobic unfavorable) contours represent 80% and 20% level contributions respectively. Yellow region at positions R_8_ and R_7_ indicates hydrophobic substituents like −CF_3_, −Ph and -C≡C- resulting in a higher activity of mGluR2 antagonist. This can be illustrated by the sudden drop in activity of chemical **14w** (pIC_50_ = 4.59, with a hydrophilic group −OH involved) comparing with **14u**, **14v**, **14x** (pIC_50_ all above 6.00).

Figures 3D and 3E depict the H-bond donor and acceptor contour maps of the CoMSIA models. Cyan color indicates the regions where H-bond donor acts as favored and purple color refers to the disfavored regions, respectively. Magenta contours encompass regions where H-bond acceptor will lead to improved biological activity, which may result in the fact that molecules with N atom at position 4 of ring D such as **8av** (pIC_50_ = 8.40), **8au** (pIC_50_ = 8.10), **8am** (pIC_50_ = 8.70), **8ao** (pIC_50_ = 8.70) generally have larger pIC_50_ value than **7z** and **8aa~8ag** molecules (pIC_50_ generally around 7.9–8.0) with position C atom at position 4. Red contours indicate an H-bond acceptor located near the red regions will result in impaired biological activity. Compound **8l** (pIC_50_ = 7.18, position B = −CF_3_) bearing lower activity than **8m** (pIC_50_ = 8.00, position B = *c*-propyl), **8n** (pIC_50_ = 8.40, position B = *i*-propyl) can be exemplified to demonstrate the disfavor for H-bond acceptor. The contour maps for H-bond donor and acceptor fields may guide the exploration of the H-bond interaction between the antagonists and the protein.

### 2.2. Results for Activity II

#### 2.2.1. 3D-QSAR Statistical Results

The dataset B composed of 69 compounds with reported experimental activities for (1S,3R)-ACPD inhibition of forskolin stimulated cAMP were divided into a training set consisting of 57 compounds and a test set containing 12 chemicals to derive the 3D-QSAR models (supporting Tables S1–S4).

For CoMFA analysis, different from activity I studies, the optimal model consists only electrostatic field descriptors with while ClogP still included in, and reveals a statistical result of *Q*^2^ = 0.503 with 6 components, *R*^2^ _ncv_ = 0.715, SEE = 0.265, *F* = 20.907. CoMFA electrostatic field accounts for 0.917 of the variance. In addition, ClogP contributes 8.3 percentages, suggesting the antagonistic activity of the mGluR2 antagonist is influenced by its hydrophobicity moderately. All five fields were employed to obtain the best CoMSIA model. The optimal model consisting of electrostatic and hydrophobic fields was generated. However, the result isn’t ideal statistically (Table 1). Therefore, the CoMFA model is mainly analyzed here.

For the optimal CoMFA model, the external test sets produced an *R*^2^ _pred_ of 0.723, which is approximate to *R*^2^ _ncv_ (0.715) for the training sets. This potently validates the predictive ability of the CoMFA model established. Figure 1C depicts the correlations between the experimental and the predict activities for both the training and test sets for the CoMFA model. A good agreement between the predicted activities and experimental data was observed from the plot.

#### 2.2.2. Contour Maps for Activity II

The optimal CoMFA model is selected to construct the “stdev*coeff” contour maps to view the field effects on the target features.

**Figure 4:**
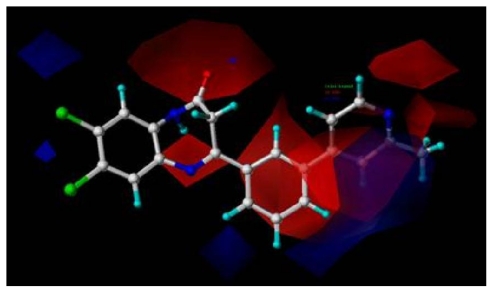
CoMFA electrostatic contour map for activity II, in combination with compound **8av**. Red contours indicate regions where negative charged groups increase activity; blue contours indicate regions where positive charged groups increase activity.

The maps generated depict regions having scaled coefficients 80% (favored) or 20% (disfavored). To aid in visualization, compound **8av** as the most active mGluR2 antagonist in the series, is shown superimposed with the CoMFA contour map (Figure 4).

Figure 4 shows the electrostatic contour maps obtained from the CoMFA analysis. The large red polyhedrons around the O and N atoms of ring II indicate the favor of electronegative charged substituents for the synthesis of potent antagonists. Similar to the electrostatic contour maps obtained from activity I, a large blue contour partially encompasses the ring D, which indicates a disfavor for electronegative substituents in this region, is observed. Compared with **8am**, **8an**, **8ao** (pIC_50_ all above 8.30), compound **8ap** (pIC_50_ = 7.82) suffers a sudden drop of potency, due to their strong electronegative atom O. The small red isopleths above the N atom in position 4 of the ring suggest that substituents should be electron rich for high binding affinity with protein. This explains the high pIC_50_ value of **8am~8ax** (with N atom at position 4 of ring D), comparing with **7z** and **8aa~8ag** (with C atom at position 4).

### 2.3. Homology Modeling Results and Docking Study

In order to find the optimal orientation of the ligand in the binding pocket of the protein, docking studies were also attempted. However, a complete validated crystal structure for mGluR2 has not yet been established. Thus, a homology model must be built based on the amino acid sequence, with a proper protein with similar structure and sequence as template. Based on chain B of template 2e4u (obtained from protein data bank), a comparative model of mGluR2 with a high sequence identity of 65.4% covering regions 23 to 558 of a total of 872 amino acids (data shown in Figure S3 contained by support information) was successfully created. The model established contains an orthosteric site, which is the active site of the protein. But according to the study of Woltering T.J., the orthosteric site is not the binding site of the series of antagonist we investigated [12]. Instead, an allosteric site which locates in the 7 transmembrane (7TM) domain of mGluR2, maybe the binding site of the antagonist due to its non-competitive nature. The series of antagonists have been reported a high affinity for allosteric site [12]. Furthermore, instead of a common allosteric site, multiple allosteric sites covering a larger binding region is suggested for mGluR2 negative allosteric modulators such as the antagonists we focus on. Their binding region is defined as the inward-facing top half of the 7TM helices [5]. Unfortunately, according to the latest sequence reported by H.Y Zhou, the 7TM region happened to be in region 554 to 872, which is not contained in modeled residue range [19].

Using bovine Rhodopsin crystal structures 1F88 and 1GZM, which is also a transmembrane protein, to build TM structure of GPCRs (G protein-coupled receptor) like mGluR1 and mGluR5, is applied recently [20–22]. Thus we attempted to build mGluR2 homology model based on 1F88 and 1GZM. In the present work, several homology models are built using this potentially effective template by a variety of modeling tools including the Swiss Model, Modweb, M4T, ESyPred3D and Modeller, but only receiving identities below 20% (data not shown), which made the model statistically insufficiently to be a valid mGluR2 structure. This may result from the low sequence identity (less than 20%) shared by different classes of GPCR, despite of their common confirmation of a hepta-helical architecture in the mGluR transmembrane [5]. The homology model reveals low sequences identity and the unideal docking results. Thus to further investigate into the structure-activity relation of the series of antagonists, pharmacophore models were established based on the two activities.

### 2.4. Pharmacophore Modeling

Presently, models for mGluR2 antagonists is not developed yet, though pharmacophore models of mGluR2 agonists have been established [23]. Herein, prior to quantitative pharmacophore development, a common-feature pharmacophore modeling study was conducted in order to identify the features required for effective mGluR2 antagonists.

DiscoTECH was employed to generate a collection of pharmacophore queries from a series of compounds some or all of which are active against a particular biological target such that all or most of active compounds satisfy the queries. Presently, 50 highly active compounds participated in the establishment of pharmacophore model for the two activities separately. The most active compounds for activity I (**8ao**) and for activity II (**8av**) were selected as reference molecules for the two pharmacophore models respectively. The maximum number of conformers generated for each compound was 50, and seven conformers were then selected.

#### 2.4.1. Pharmacophore Model for Activity I

**Figure 5:**
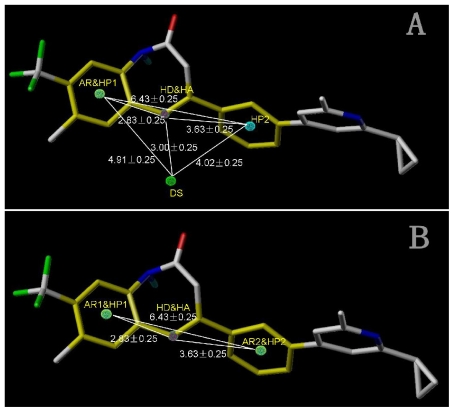
The pharmacophoric features derived of (**A**) model_001 for activity I with six features generated; and (**B**) model_002 for activity I with six features generated presented in template molecule **8ao**, respectively. AR represents aromatic center; HP refers to hydrophobic center; HD and HA are short for H-bond donor and acceptor respectively; DS refers to H-bond donor site. The distances (Ǻ) between these sites are marked in white lines.

In total, nine models (as shown in Table 2) were generated using the most active 50 antagonists with no omitting for activity I studies. Model_001 (Figure 5A) was selected for subsequent studies despite its second highest score value (1.8562) because it has useful features of a higher diversity compared with model_002 (Figure 5B), which possesses the highest score (2.3577).

Figure 5A shows the optimal pharmacophore model (model_001) obtained with a score of 1.8562 and tolerance distance of 0.25 Å. The model has six essential features required for high receptor binding affinity, which contain two hydrophobic sites (HP1 and HP2), an H-bond donor atom (HD), an H-bond donor site (DS), one H-bond acceptor atom (HA) and an aromatic center (AR). The distances between these pharmacophoric features are listed in Table 3. Two aromatic ring (ring I and ring III), a heterocyclic ring (ring II) containing N and a constrained conformation features are common in all the 50 chemicals. The hydrophobic center and the planar group serve as the rigid portion of the molecular scaffold that satisfies the overall geometric and steric requirements of binding.

This model has the characteristic features required for an ideal pharmacophoric query, because it possesses the important interactions required for this series of antagonists and was consistent with previously reported scaffold of potent antagonists [12]. Closer inspection of the pharmacophore model reveals that H-bond donor and H-bond acceptor atoms were in agreement with the results from the CoMSIA model. To be considered as a hit, a compound has to fit all the features of the pharmacophore model consequently.

#### 2.4.2. Pharmacophore Model for Activity II

**Figure 6:**
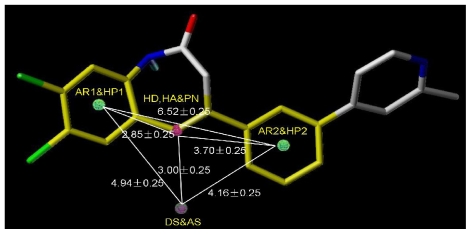
The pharmacophoric features derived of model_001 for activity II with nine features generated presented in template molecule **8av**. AR represents aromatic center; HP refers to hydrophobic center; HD and HA are short for H-bond donor and acceptor respectively; PN refers to positive N; DS and AS represent H-bond donor site and acceptor site respectively. The distances (Ǻ) between these sites are marked in white lines.

For activity II studies, based on 50 compounds of high antagonistic activity, only one model was obtained with a score of 1.8286 (as shown in Table 4) and tolerance of 0.25 Å. Nine essential features are observed in this model required for the high antagonistic activity, including two hydrophobic sites (HP1 and HP2), an H-bond donor atoms (HD), one H-bond donor site (DS), one H-bond acceptor site (AS), one H-bond acceptor atom (HA), one positive N (PN) and two aromatic centers (AR1 and AR2). Figure 6 shows the pharmacophoric features generated in this model, with the most active compound **8av** for activity II as reference. The distances between these pharmacophoric features are listed in Table 5.

An additional positive N, an acceptor site and an aromatic center are generated besides those similar features shared with the pharmacophore model generated previously for activity I, indicating a necessity of the presence of positive N and acceptor atom for the synthesis of potent mGluR2 antagonists that perform well in cAMP test. Compounds contain all the nine features of the pharmacophore model are supposed to be more capable of performing antagonistic activity towards mGluR2.

For both activity I and II studies, pharmacophore features derived are in range of the tricycle scaffold. This indicates that the basic scaffold features participate significantly in the pharmacologic effect of the antagonist. Using the models developed, a new effective scaffold that contains these phamacophore features can be synthesized. In addition, CoMFA and CoMSIA contour maps derived denote the favorable features of substituents. With a combination of phamacophore features and CoMFA, CoMSIA contour maps, new potent antagonists can be designed.

### 2.5. Comparison between the Two Activities

**Figure 7:**
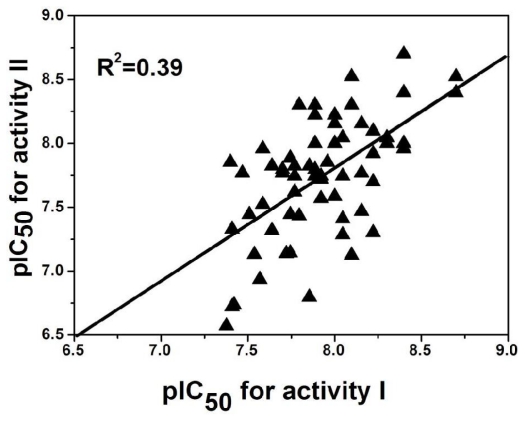
A correlation plot of activities for activity I (affinity test: partial displacement of [^3^H]-LY354740) and activity II (cell based test: inhibition of effect of1S,3R-ACPD on cAMP level).

Out of the 137 compounds employed in the investigation, 69 have both antagonistic activity I and II. To explore the possible relationship of the two potencies, a mathematical model is established and its corresponding plot using activity I and II as the X and Y coordinate respectively is drawn (Figure 7) based on these molecules. Clearly, a good linear correlationship is observed between the two activities with a correlation coefficient of *R*^2^ = 0.39. This may suggests that the activities of these antagonist in radioligand binding studies (Rat mGluR2 [^3^H]-LY354740 binding, activity I) and in functional Cyclase Inhibition Assay (cAMP assay, activity II), though differ considerably, correlate with each other to a significant extent. The prediction that the binding ratio reflected the functional efficacy of a compound was also supported by measurement of the ability of a number of compounds acting at dopamine receptors to inhibit rD2(444)-mediated inhibition of cyclic AMP production [24]. However, the pIC_50_ value of the two activities still differs from each other, due to the different mechanism of cAMP production and binding assay: Radioligand binding studies and cAMP assay are two strategies in drug screening technology to investigate the activity of a ligand. cAMP assay is based on a second messenger and assesses the antagonistic activity of a ligand through the level of cAMP. That enables us to appreciate the molecular features of inhibition by the regulatory subunits as well as the activation by cAMP [25]. Radioligand binding studies do not involve the signal path, but directly investigate into the interaction between the radiolabelled ligands and the receptor. The first objective in binding assay is to ensure that the binding equilibrium is reached. Then K_D_, a parameter to describe the affinity of a ligand for a receptor can be calculated [26].

In general, CoMFA and CoMSIA statistic results for the two activities differ in several aspects. (I) The best models for activity I employ all field descriptors in both CoMFA and CoMSIA (steric and electrostatic in CoMFA and steric, electrostatic, hydrophobic, H-donor, H-acceptor in CoMSIA) analyses. While for activity II, optimal model contains only electrostatic field in CoMFA analysis; (II) Steric field exerts deeper influence on activity I rather than activity II, for in activity I study, it contributes considerable percentages in both CoMFA (48.8%) and CoMSIA (11.2%) analysis. However, for activity II study, neither the best CoMFA model nor the best CoMSIA model employs steric field as a contributor to the variance of the activity; (III) Electrostatic field clearly plays a dominant role in the variance of activity II, while for activity I, more factors including steric field, hydrophobic field, H-donor and H-acceptor field contribute to the variance of activity of the series of antagonists; (IV) The hydrophobicity of a antagonist exerts more influence on its activity II rather than activity I for two reasons. Firstly, hydrophobic field plays more essential role in the later one on improving the value of statistical parameters (*Q*^2^, *R*^2^ _ncv_, *R*^2^ _pre_) that are crucial for evaluating the reliability of the model. Thus, in the best CoMSIA models, hydrophobic field contributes a higher percentage to activity II (40.9%). Furthermore, ClogP, which is a well established measure of the compound’s hydrophobicity. Thus, it contributes more in cAMP functional assay (activity II), with 8.3% (activity II) over 5.1% (activity I) in CoMFA and 13.6% (activity II) over 3.5% (activity I) in CoMSIA. These differences in statistic result may result from the difference in mechanism of the two experiments revealing the activity of the antagonist.

From the differences above, we may conclude that factors like the steric feature, electrostatic feature, hydrophobicity of a function group all need to be considered to best improve the performance of the antagonists in radioligand binding assay. However, simply confusing on the electrostatic character as well as the hydrophobicity of the substituents may help design drugs showing potent antagonism in cAMP assay.

Analysis of the 3D-QSAR contour maps derived from activity I and activity II suggest that they generally have very similar structural requirements for potent ligands. The electrostatic contour maps all indicate that electropositive groups above N atom at position 4 of ring D will benefit the potency of the antagonist. Moreover, a large blue contour partially encompassing the pyridine ring is observed in all four of the contour maps. Still, a subtle difference exists: electropositive groups above the O atom of ring II decrease the activity I while favor the activity II. Also, large red contour around the N atom of ring II suggests a favor for electropositive substituents or atoms. These differences and similarities in molecular structural features of potent antagonists may help increasing or reducing the activity of the compound by changing its substituents.

In addition, the pharmacophore models derived from the two activities generally share the same features, excepting for a few difference. An H-donor atom, an H-donor acceptor atom, an H-donor site, two hydrophobic centers and an aromatic ring are common features for the two models. Besides, for pharmacophore model of activity II, a positive N, an H-bond acceptor site and an additional aromatic ring are featured as essential characters of potent drugs. Thus, here the active sites shared by the two models are considered fundamental pharmacophore features for potent mGluR2 antagonists. The highly similarity of the two models may reflect the predict ability of radioligand binding assay towards pharmacophore function of the antagonists. These models can be used as guidance for the design of new mGluR2 antagonists of high activity and selectivity.

## 3. Material and Methods

### 3.1. Dataset and Biological Activity

A total of 137 8-ethynyl-1,3-dihydro-benzo[b][1,4]diazepin-2-one derivatives with common characteristics of inhibiting the [^3^H]-LY354740 binding to rat mGluR2 receptors were adopted as dataset A to build models for activity I studies. Compound 4, which has identical structure but different activity statistics than **7p**, was discarded from the dataset as suspected error statistic. In addition, compound **3** and **15o** are identical both in structure and statistics, and **15o** is adopted. Also, for activity II, 69 among the 137 compounds with a statistically reported function of inhibiting the forskolin stimulated cAMP production were adopted as dataset B to build models. The statistics were collected from the experimental values of Woltering T.J. *et al*. [12]. The original names of compounds in the four articles are preserved. The in vitro biological activities of these compounds were converted into the corresponding pIC_50_ (−log IC_50_) values, which were used as dependent variables in the 3D-QSAR analyses. PIC_50_ values and structures of 25 typical molecules are shown in Table 6. In approximately a ratio of 4:1, the whole data set was divided into training (110 molecules) and test (27 molecules) sets in models based on activity I and training (57 molecules) and test (12 molecules) sets in models for activity II, respectively. With a desired function of representing the entire dataset to the most, the test set chemicals were picked considering several criterions: First and foremost, their pIC_50_ values are randomly but uniformly distributed in the range of the values for the whole set. Furthermore, the collection of their structures is typical enough to represent the entire dataset. The training and test set are listed in Tables S1–S4 (supporting information). All molecular modeling and 3D-QSAR studies were performed using the SYBYL6.9 molecular modeling software package (Tripos Associates, St. Louis, MO, USA). Partial atomic charges were calculated by the Gasteiger-Huckel method [27], energy minimization and conformational search were performed using Tripos molecular mechanics force field [28] by conjugating method with a convergence criterion of 0.001 kcal/mol. To obtain relatively stable conformation, the energy gradient limit was set at 0.05 kcal/mol Å. Table S1–S4 (Supporting Information) lists all the structures and biological values (pIC_50_) of the dataset, and the representative skeletons and pIC_50_ values are depicted in Table 6.

### 3.2. Conformational Sampling and Alignment

**Figure 8:**
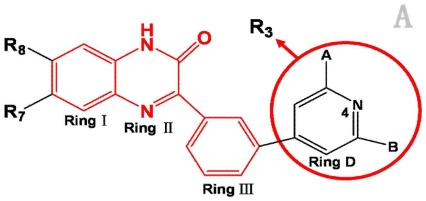

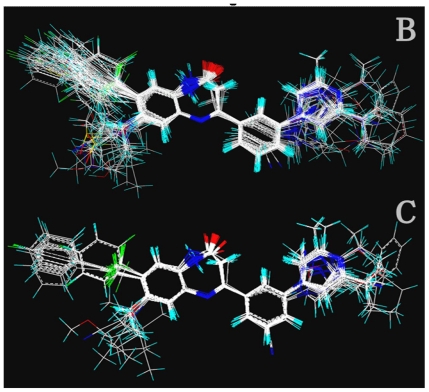
Scaffold of the mGluR 2 antagonists and molecular alignment of compounds for two models. (**A**) Common substructure of the molecules is shown in red with 8ao as template molecule, and the heterocyclic ring (not commonly shared) as a substituent in position R_3_ is named ring D to better describe some compounds in group 8; (**B**) For activity I, molecular alignment using 137 molecules of dataset A; (**C**) For activity II, alignment model using 69 compounds of dataset B.

Molecular alignment of compounds, which align all the compounds together by common scaffold, is a key step in the process of establishing 3D-QSAR models [29]. In the alignment, a molecule with relatively high biological activity and fairly fixed conformation is usually adopted as template. In this work, the most active compounds for the two activities: **8ao** (activity I, pIC_50_ = 8.7) and **8av** (activity II, pIC_50_ = 8.7) were used as template molecules in the two models respectively. The tricycle which they share with other molecules was chosen as the common scaffold. Figure 8A describes the common substructure for the alignment which is marked in red. A ring in position R_3_ was named ring D and positions A, B and 4 are shown in the figure. Based on an atom-by-atom superimposition principle, the ligand-based alignment of the molecules was carried out by using substructure-alignment function available in SYBYL. Figure 8B and Figure 8C show the resulting ligand-based alignment model of activity I and activity II respectively.

### 3.3. CoMFA and CoMSIA Field Calculation

CoMFA approach, proposed by Cramer and co-workers in 1988, describes the molecular properties by 3D steric (Lennard–Jones) and electrostatic (Coulomb) fields, evaluated over a lattice of points. In a similar approach described as the (CoMSIA) by Klebe and co-workers in 1994, a probe atom is used to calculate the similarity indices, at regularly spaced grid points, for the pre-aligned molecules.

In CoMFA calculations, the aligned training set molecules were placed in a 3D grid box. The steric and electrostatic field energies were calculated using sp^3^ carbon as probe atom. The energies were truncated to 30 kcal/mol. CoMFA method only calculates the steric and electrostatic interactions, yet CoMSIA not only calculates the steric and electrostatic interactions, but also calculates the hydrophobic, HB donor and HB acceptor interactions [30]. The basic assumption of CoMSIA is that a suitable sampling of the steric, electrostatic, hydrophobic and HB acceptor interactions generated around a set of aligned molecules with a probe atom might provide all important features for understanding their biological activities, and that the changes in binding affinities of ligands are related to changes in molecular properties [31]. Similar to CoMFA studies, the CoMSIA method employs a 3D lattice with regular grid points separated by 2Å to place aligned molecules. CoMSIA uses a Gaussian-type distance-dependent function to assess five fields of different physicochemical properties. The default value of 0.3 was used as attenuation factor [18,32]. Because of the different shape of the Gaussian function, CoMSIA similarity indices (A_F_) for a molecule j with atom i at a grid point q are calculated by Equation 1 as follows:

(1) $$A_{F,k}^{\text{q}}(j)=-\sum \omega_{probe,k}\omega_{ik}e^{-\alpha r_{iq}^{2}}$$

where *ω*_probe,k_ is the probe atom with radius 1Å, charge +1, hydrophobicity +1, hydrogen bond donating +1 and hydrogen bond accepting +1. *ω*_ik_ is the actual value of the physicochemical property *k* of atom i. *r*_iq_ is the mutual distance between the probe atom at grid point q and item i of the test molecule [33].

Furthermore, the logP value of a compound, which is the logarithm of its partition coefficient between *n*-octanol and water log (*c*_octanol_/*c*_water_), is a well established measure of the compound’s hydrophilicity. In the present work, Clog P (calculated logP) was used as an additional descriptor in the CoMFA and CoMSIA analysis to study the effects of lipophilic parameters on activity.

### 3.4. Partial Least Square Analysis

Partial least-squares (PLS) methodology implemented in the QSAR model of SYBYL was used in deriving the 3D-QSAR models. This method replaces the original variables by a small set of linear combinations and is a variation of principal component regression [34,35]. In this work, PLS was used to analyze the training set by correlating the variation in their pIC_50_ values (the dependent variable) with variations in their CoMFA/CoMSIA interaction fields (the independent variables). Initially, cross-validation analysis was accomplished with the leave-one-out (LOO) methodology, where one compound was excluded from the original dataset and its activity was predicted by the new model derived from the rest of the database. That gives the LOO-CV (*R*^2^) as a statistical index of predictive power. Then a non-cross-validation analysis was carried out to calculate the Pearson coefficient and the standard error of estimates (SEE).

During PLS process, several statistical parameters including the *Q*^2^ and the above *R*^2^ _ncv_ are crucial for evaluating the reliability of the model generated. As a cross-validated coefficient, *Q*^2^ is used as a statistical index of the predictive power of the model, and is calculated by Equation 2 where the Y_predicted_, Y_observed_ and Y_mean_ are predicted, actual and mean values of the target property, respectively [36].

(2) $$q^{2}=1-\frac{\sum_{Y}{\left(Y_{predicted}-Y_{observed}\right)}^{2}}{\sum_{Y}{\left(Y_{observed}-Y_{mean}\right)}^{2}}$$

In order to evaluate the real predictive ability of the best models generated by the CoMFA/CoMSIA analyses, 27 compounds are treated as the external validation set for activity I and 12 compounds for activity II. A predictive R value was then obtained with Equation 3:

(3) $$r_{pred}^{2}=1-PRESS/SD$$

Where SD denotes the sum of squared deviation between the biological activities of the test set molecule and the mean activity of the training set molecules, PRESS represents the sum of squared deviations between the experimental and predicted activities of the test molecules [37].

### 3.5. DISCOtech Analysis

3D pharmacophore mappings based on distance comparison technique were derived from the 50 most active compounds for activity I and activity II respectively. Pharmacophore models in particular involve the identification of the pharmacophoric pattern common to a set of known actives and the use of this pattern in a subsequent search. DISCOtech™, a well established module for designing pharmacophoric maps and frequently used in the process of virtual screening to discover new leads, is employed in the establishment [38,39]. DISCOtech identifies features that could be elements in a pharmacophore model from a set of molecules that binds to a common binding site [40,41]. These features include hydrogen bond donor atoms (HD), hydrogen bond acceptor atoms (HA), hydrogen donor (DS) and acceptor site (AA), charge centers, centers of mass of hydrophobic rings (HP), aromatic rings (AR) and positive N (PN). In this study, a stochastic search method was used to create conformers for the molecules. The maximum number of conformers generated for each compound was 50, and seven conformers were then selected. The 50 most active molecules were employed with most active antagonist selected as reference compound. Min 4 and Max 16 features were allowed to find during the analysis. Tanimoto threshold was set as 0.6. All other parameters were retained as default values. The best DISCOtech pharmacophore model with relatively high score, more useful features and moderate pairwise tolerance was proposed.

## 4. Conclusions

In this paper, the 3D-QSAR studies and ligand-based modeling of 137 1,3-dihydrobenzo[ b][1,4]diazepin-2-one derivatives were, for the first time, performed using CoMFA and CoMSIA tools. In 2008, a Chinese paper reported a model concerning affinity test using 30 compounds in the first one among the series of article [42]. Two series of models were built using statistics from affinity assay (activity I) and cell test (activity II) respectively. The constructed 3D-QSAR models exhibited proper predictive powers in both the internal and external tests. The resulting contour maps produced by the models provide a platform for the screening of novel inhibitors and enables the interpretation of their binding models to mGluR2. A good consistency between the CoMFA and CoMSIA contour maps and the pharmacophore model was observed, proving the reliability and robustness of the models. Overall, our main findings are summarized as follows: (1) Bulky substituents in R7, R3 and position A benefit activity I of the series of derivatives, and decrease the potency when projected in R8 and position B of ring D; (2) Hydrophilic groups at position A and B of Ring D may increase the antagonistic activity I; (3) Electrostatic field plays an essential role in improving the antagonism of the compounds performed in cAMP assay (activity II); (4) An H-donor atom, an H-donor acceptor atom, an H-donor site, two hydrophobic centers and an aromatic ring are shared pharmacophoric features of the two models. Besides, for pharmacophore model of activity II, a positive N, an H-bond acceptor site and an additional aromatic ring are featured as essential characters of potent drugs. In addition, the amino acid sequence of the human mGluR2 receptor consists of 872 residues and shows a sequence identity of 97% to the amino acid sequence of rat mGluR2, besides highly similar anatomy, thus made the model highly applicable to create human mGluR2 antagonists [43]. All the correlation of the results obtained from above QSAR and pharmacophore studies, we hope, may lead to a better understanding of the structural requirements for enhanced activity and help in the design of new and more potent mGluR2 antagonists.

## Supplementary Information



## Figures and Tables

**Table 1:** Summary of **comparative molecular field analysis (**CoMFA) and **comparative molecular similarity analysis** CoMSIA results for activity I/activity II.

PLS Statistics	Activity I		Activity II	
	
	CoMFA	CoMSIA	CoMFA	CoMSIA
*Q*^2^	0.513	0.450	0.503	0.367
*R*^2^_ncv_	0.868	0.899	0.715	0.657
SEE	0.296	0.273	0.265	0.285
*F*	96.022	101.353	20.907	24.927
*R*^2^_pre_	0.876	0.735	0.723	0.667
SEP	0.288	0.420	0.241	0.265
OPN	7	8	6	4
*Contribution*				
Steric	0.488	0.112		
Electrostatic	0.461	0.277	0.917	0.455
Hydrophobic		0.184		0.409
H-donor		0.135		
H-acceptor		0.256		
Clogp	0.051	0.035	0.083	0.136

*Q*^2^, cross-validated correlation coefficient after the leave-one-out procedure; *R*^2^ _ncv_, non-cross-validated correlation coefficient; SEE, standard error of estimate; *F*, ratio of *R*^2^ _ncv_ explained to unexplained = *R*^2^ _ncv_/(1 – *R*^2^ _ncv_); *R*^2^ _pre_, predicted correlation coefficient for the test set of compounds; SEP, standard error of prediction; OPN, optimal number of principal components.

**Table 2:** Number of models obtained along with the pharmacophoric features and tolerance values for each of the DISCO pharmacophoric run for activity I.

MODEL	SIZE a	HITS b	SCORE c	TOLERANCE d	DMEAN e
MODEL_002	6	50	2.3577	0.25	3.4348
MODEL_001	6	50	1.8562	0.25	3.4163
MODEL_005	7	50	1.7220	0.25	3.0344
MODEL_009	7	50	1.3646	0.25	3.4012
MODEL_003	7	50	1.3629	0.25	3.3955
MODEL_006	7	50	1.3607	0.25	3.3880
MODEL_008	6	50	0.6783	0.25	2.0754
MODEL_004	6	50	0.3735	0.25	2.8621
MODEL_007	6	50	0.3733	0.25	2.8616

a SIZE, number of features in the model;

b HITS, number of molecules that matched during the research;

c SCORE, an overall measure of fit and of overlap for the entire collection of structure;

d TOLERANCE, initial tolerance setting (from 0.25 to 2.5);

e DMEAN, average inter-point distance.

**Table 3:** Relative intramolecular distances between pharmacophoric feature points for model_001 of activity I (Ǻ).

	AR1	HP1	DS	HP2
HA	2.83	2.83	3.00	3.63
HD	2.83	2.83	3.00	3.63
DS	4.91	4.91		4.02
HP2	6.43	6.43	4.02	

AR represents aromatic center; HP refers to hydrophobic center; HD and HA are short for H- bond donor and acceptor respectively; DS refers to H-bond donor site.

**Table 4:** Number of models obtained along with the pharmacophoric features and tolerance values for each of the DISCO pharmacophoric run for activity II.

MODEL	SIZE a	HITS b	SCORE c	TOLERANCE d	DMEAN e
MODEL_001	9	50	1.8286	0.25	3.3280

a SIZE, number of features in the model;

b HITS, number of molecules that matched during the research;

c SCORE, an overall measure of fit and of overlap for the entire collection of structure;

d TOLERANCE, initial tolerance setting (from 0.25 to 2.5);

e DMEAN, average inter-point distance.

**Table 5:** Relative intramolecular distances between pharmacophoric feature points for model_001 of activity II (Ǻ).

	AR1	HP1	DS	HP2
HA	2.83	2.83	3.00	3.63
HD	2.83	2.83	3.00	3.63
DS	4.91	4.91		4.02
HP2	6.43	6.43	4.02	

AR represents aromatic center; HP refers to hydrophobic center; HD and HA are short for H-bond donor and acceptor respectively; PN refers to positive N, DS and AS represent H-bond donor site and acceptor site respectively.

**Table 6:** Representative structures and inhibitory activities (pIC_50_) of the dataset.

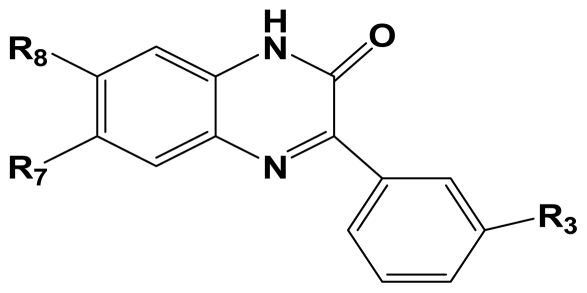					
NO.	R_3_	R_8_	R_7_	Activity I	Activity II
**1**	H	Me	H	5.1938	
**2**	CN	Ph-C≡C-	H	7.4685	7.7696
**14u**	CN	2-Thiazolyl-C≡C-	H	6.5229	
**14v**	CN	2-Pyridyl-C≡C-	H	6.0605	
**14x**	CN	H_2_C=C(Me)-C≡C-	H	6.3979	
**14aa**	CN	Ph-C≡C	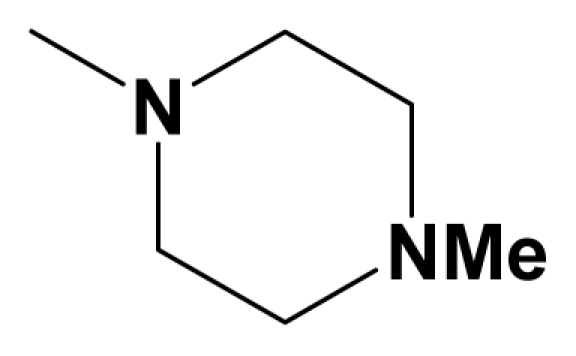	6.5560	
**15c**	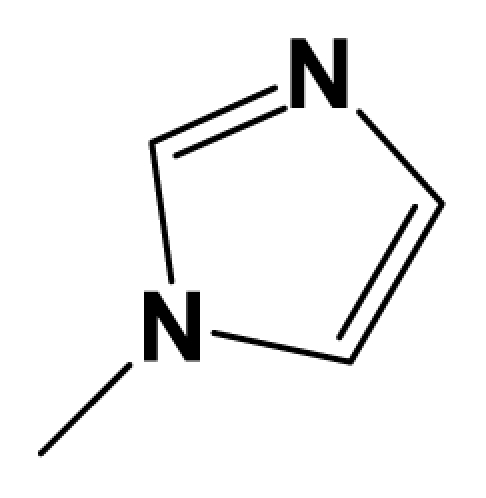	2-F-C_6_H_4_-C≡C-	H	7.6990	7.7959
**15m**	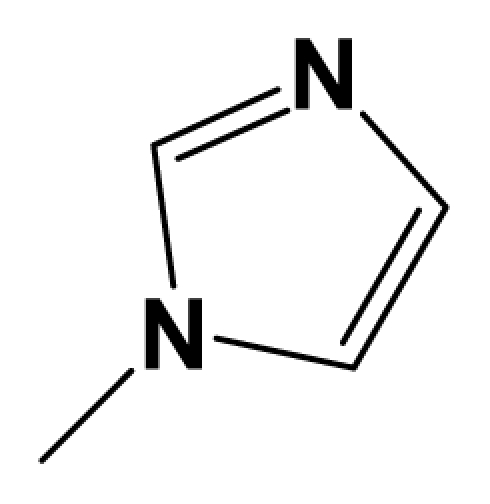	Ph-C≡C-	-OCH_2_CN	7.7447	7.8861
**15q**	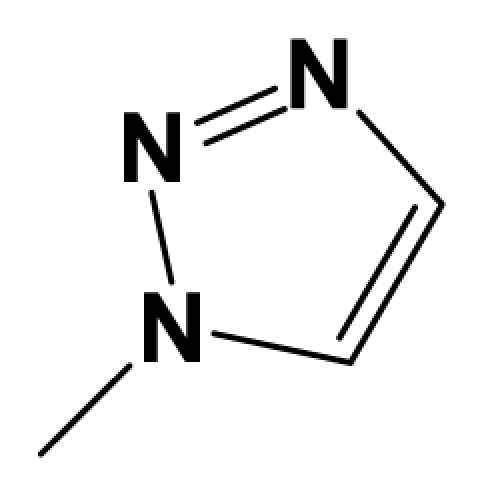	4-F-C_6_H_4_-C≡C-	-OH	7.6990	7.7696
**7g**	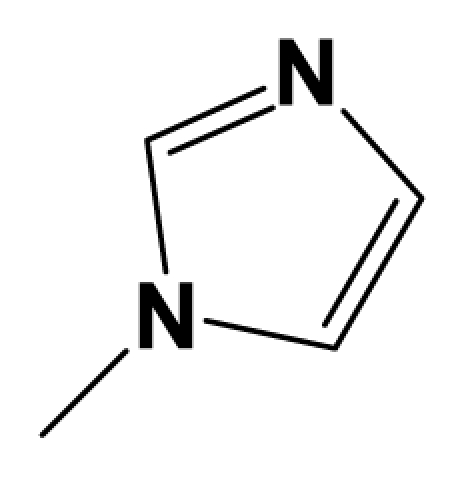	*Cyclo*-propyl	H	6.6778	
**7o**	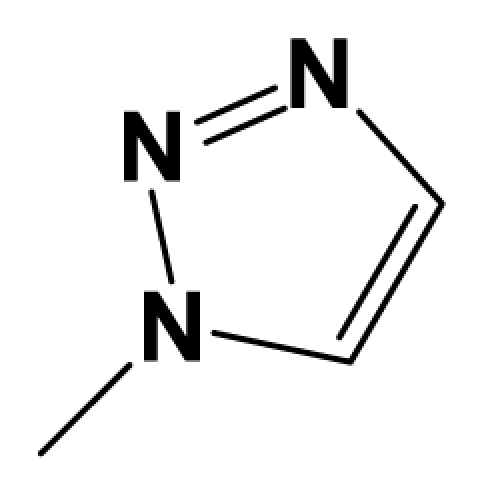	4-F-C_6_H_4_-	H	7.5086	7.4437
**7z**	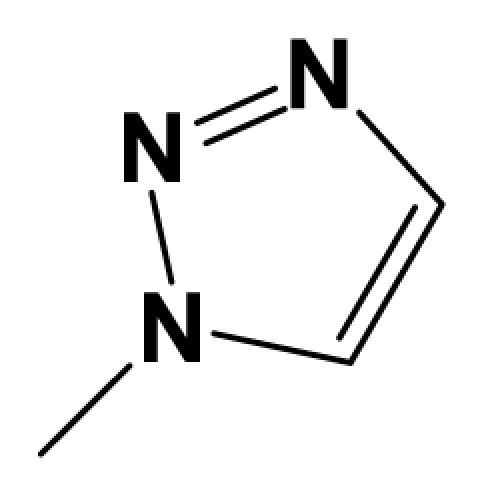	F_3_C-	*Iso*-butylN(Me)	7.9586	7.8539
**7ac**	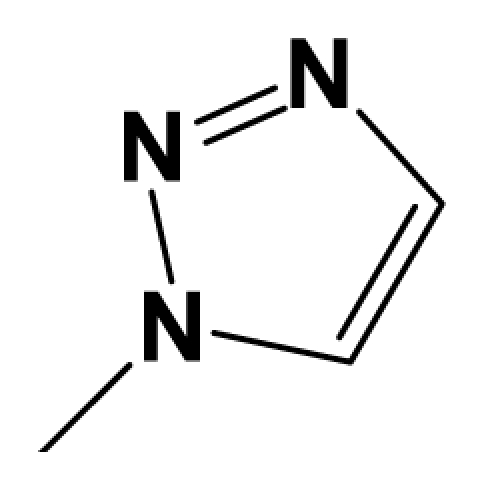	F_3_C-	MeO	7.5376	7.1308
**8a**	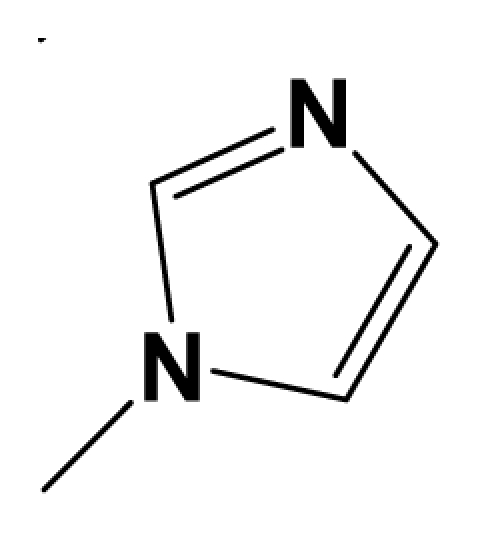	F_3_C-	Me	7.9208	7.7212
**8h**	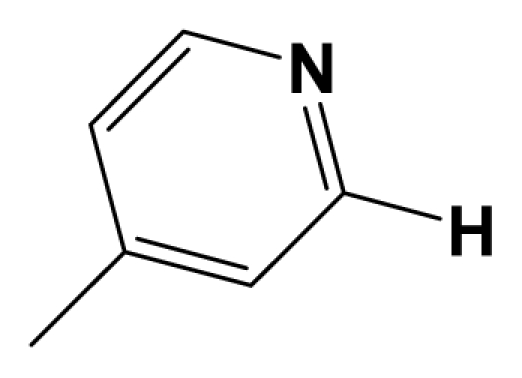	F_3_C-	Me	8.3979	7.9586
**8y**	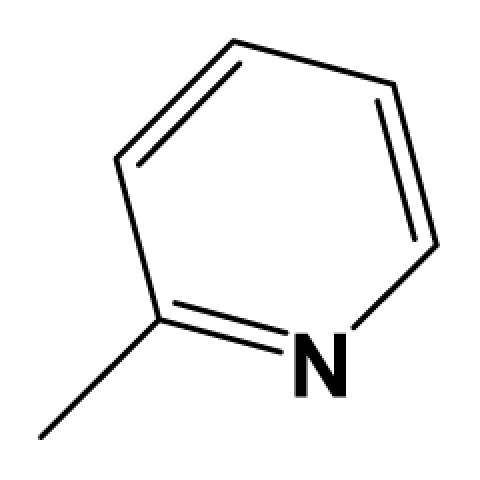	F_3_C-	Me	7.5850	7.5229
**8aa**	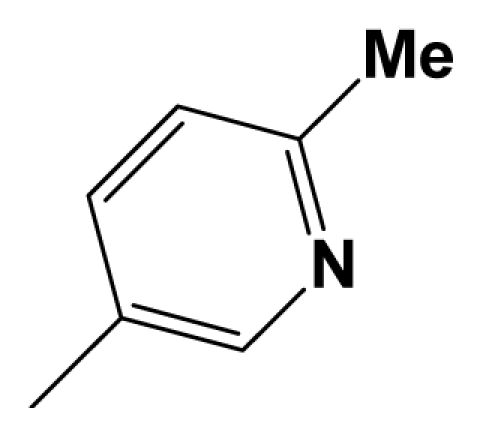	F_3_C-	Me	7.8861	8.3010
**8ae**	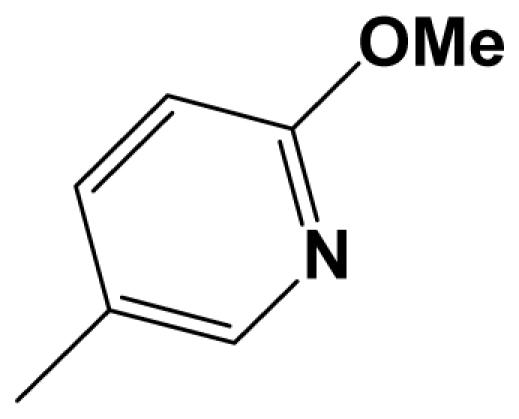	F_3_C-	Me	8.3010	8.0000
**8aj**	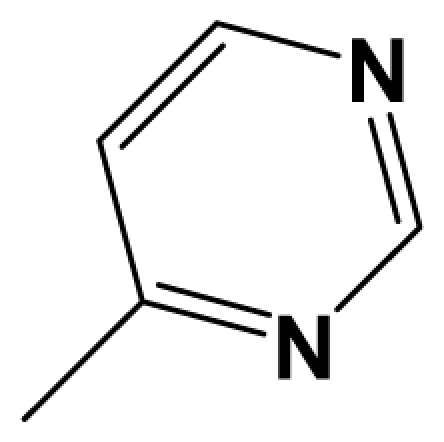	F_3_C-	Me	8.2218	7.3010
**8ao**	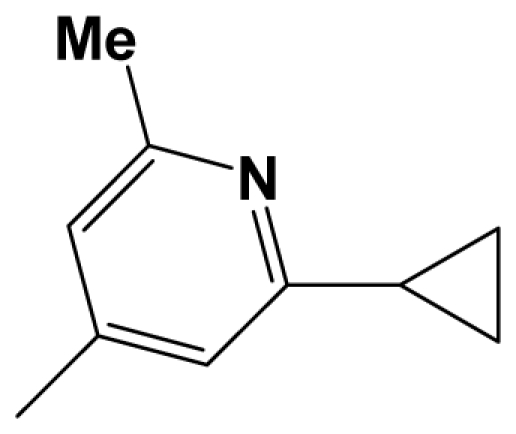	F_3_C-	Me	8.6990	8.3979
**8av**	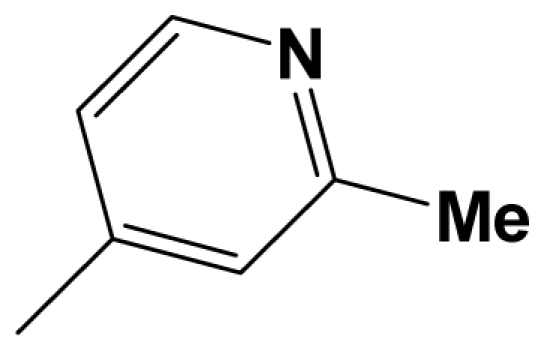	Cl	Cl	8.3979	8.6990
