# Supplementary material for: Investigation on Quantitative Structure Activity Relationships and Pharmacophore Modeling of a Series of mGluR2 Antagonists
Source: Int J Mol Sci. 2011 Sep 16;12(9):5999–6023. doi: 10.3390/ijms12095999 (PMC3189765; doi:10.3390/ijms12095999)
Supplement: Supplementary file 1 [file ijms-12-05999-s001.pdf]

# Supplementary Information

**Meng-Qi Zhang [1], Xiao-Le Zhang [2], Yan Li [1,*], Wen-Jia Fan [1], Yong-Hua Wang [3,4], Ming Hao [1], Shu-Wei Zhang [1] and Chun-Zhi Ai [3]**

[1] Department of Materials Science and Chemical Engineering, Dalian University of Technology, Dalian, Liaoning 116024, China; E-Mails: manna1989@mail.dlut.edu.cn (M.-Q.Z.); rosemarryfan@gmail.com (W.-J.F.); dluthm@yeah.net (M.H.); zswei@dlut.edu.cn (S.-W.Z.)

[2] Department of Mathematical Sciences, Dalian University of Technology, Dalian, Liaoning 116024, China; E-Mail: xlfree@foxmail.com

[3] Lab of Pharmaceutical Resource Discovery, Dalian Institute of Chemical Physics, Graduate School of the Chinese Academy of Sciences, Dalian, Liaoning 116023, China; E-Mail: aicy@dicp.ac.cn

[4] Center of Bioinformatics, Northwest A&F University, Yangling, Shaanxi 712100, China; E-Mail: yhwang@dlfu.edu.cn

* Author to whom correspondence should be addressed; E-Mail: yanli@dlut.edu.cn; Tel. +86-411-84986062; Fax: +86-411-84986063.

*Received: 2 August 2011; in revised form: 1 September 2011 / Accepted: 9 September 2011 / Published: 16 September 2011*

---

**Abstract:** MGluR2 is G protein-coupled receptor that is targeted for diseases like anxiety, depression, Parkinson's disease and schizophrenia. Herein, we report the three-dimensional quantitative structure–activity relationship (3D-QSAR) studies of a series of 1,3-dihydro-benzo[b][1,4]diazepin-2-one derivatives as mGluR2 antagonists. Two series of models using two different activities of the antagonists against rat mGluR2, which has been shown to be very similar to the human mGluR2, (activity I: inhibition of [$^3$H]-LY354740; activity II: mGluR2 (1*S*,3*R*)-ACPD inhibition of forskolin stimulated cAMP.) were derived from datasets composed of 137 and 69 molecules respectively. For activity I study, the best predictive model obtained from CoMFA analysis yielded a $Q^2$ of 0.513, $R^2_{ncv}$ of 0.868, $R^2_{pred}$ = 0.876, while the CoMSIA model yielded a $Q^2$ of 0.450, $R^2_{ncv}$ = 0.899, $R^2_{pred}$ = 0.735. For activity II study, CoMFA model yielded statistics of $Q^2$ = 0.5, $R^2_{ncv}$ = 0.715, $R^2_{pred}$ = 0.723. These results prove the high predictability of the models. Furthermore, a combined analysis between the CoMFA, CoMSIA contour maps shows that: (1) Bulky substituents in $R_7$, $R_3$ and position A benefit activity I of the antagonists, but decrease it when projected in $R_8$ and position B; (2) Hydrophilic groups at position A and B increase both antagonistic activity I and II; (3) Electrostatic field plays an essential rule in the variance of activity II. In search for more potent mGluR2 antagonists, two pharmacophore models were developed separately for the two activities. The first model reveals six pharmacophoric features, namely an aromatic center, two hydrophobic centers, an H-donor atom, an

H-acceptor atom and an H-donor site. The second model shares all features of the first one

and has an additional acceptor site, a positive N and an aromatic center. These models can be used as guidance for the development of new mGluR2 antagonists of high activity and selectivity. This work is the first report on 3D-QSAR modeling of these mGluR2 antagonists. All the conclusions may lead to a better understanding of the mechanism of antagonism and be helpful in the design of new potent mGluR2 antagonists.

**Keywords:** 3D-QSAR; mGluR2 antagonist; CoMFA; CoMSIA; pharmacophore modeling

---

**Table S1.** Structure–activity relationship data related to the series of 1,3-dihydro-benzo[b][1,4]diazepin-2-one derivatives described in this study (group 14).

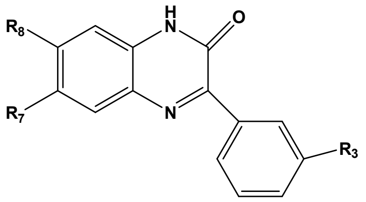

| NO | $R_3$ | $R_8$ | $R_7$ | Activity I ($pIC_{50}$) | Activity II ($pIC_{50}$) |
|---|---|---|---|---|---|
| **1**# | H | Me | H | 5.1938 | |
| **2**§ | CN | Ph-C≡C- | H | 7.4685 | 7.7696 |
| **14a** | MeO | Me | H | 5.1818 | |
| **14b** | Cl | Me | H | 5.5498 | |
| **14c**# | $CF_3$ | Me | H | 5.8153 | |
| **14d** | CN | Me | H | 5.5376 | |
| **14e**# | H | Ph-C≡C- | H | 7.5850 | |
| **14f** | MeO | Ph-C≡C- | H | 6.0168 | |
| **14g** | Cl | Ph-C≡C- | H | 7.0969 | |
| **14h** | $CF_3$ | Ph-C≡C- | H | 6.5719 | |
| **14i** | I | Ph-C≡C- | H | 6.8125 | |
| **14j** | $CONH_2$ | Ph-C≡C- | H | 6.4461 | |
| **14k** | 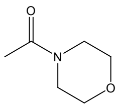 | Ph-C≡C- | H | 6.4437 | |
| **14l** | 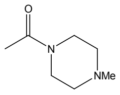 | Ph-C≡C- | H | 5.9830 | |
| **14m** | CN | Ph-C≡C- | H | 7.4685 | |
| **14n**# | CN | 2-Cl-$C_6H_4$-C≡C- | H | 7.1192 | |
| **14o** | CN | 4-Me-$C_6H_4$-C≡C- | H | 7.1938 | |
| **14p**# | CN | 4-MeO-$C_6H_4$-C≡C- | H | 7.1675 | |
| **14q** | CN | 4-F-$C_6H_4$-C≡C- | H | 7.4685 | |
| **14r** | CN | 2-F-$C_6H_4$-C≡C- | H | 7.5229 | |

**Table 1S.** *Cont.*

| | | | | |
|---|---|---|---|---|
| **14s** | CN | 2,4-di-F-$C_6H_4$-C≡ C- | H | 6.3188 |
| **14t** | CN | 2-Thiophenyl-C≡C - | H | 7.4949 |
| **14u** | CN | 2-Thiazolyl-C≡C- | H | 6.5229 |
| **14v** | CN | 2-Pyridyl-C≡C- | H | 6.0605 |
| **14w** | CN | $HO(Me)_2C$-C≡C- | H | 4.5850 |
| **14x** | CN | $H_2C$=C(Me)-C≡C- | H | 6.3979 |
| **14y** | CN | Ph-C≡C- | $-NMe_2$ | 7.2218 |
| **14z** | CN | Ph-C≡C- | 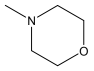  | 6.4225 |
| **14aa**[#] | CN | Ph-C≡C- | 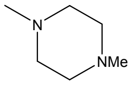  | 6.5560 |
| **14ab** | CN | Ph-C≡C- | 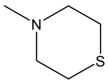  | 7.3979 |
| **14ac**[#] | CN | Ph-C≡C- | 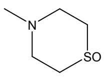  | 6.3507 |
| **14ad**[#] | CN | Ph-C≡C- | 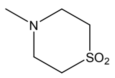  | 6.7959 |
| **14ae** | CN | Ph-C≡C- | 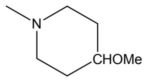  | 7.0862 |
| **14af** | CN | Ph-C≡C- | -OMe | 7.0362 |
| **14ag**[#] | CN | Ph-C≡C- | $-OCH_2CH_2OMe$ | 7.5528 |
| **14ah** | CN | Ph-C≡C- | $-OCH_2CO_2H$ | 6.2218 |
| **14ai** | CN | Ph-C≡C- | $-OCH_2CONH_2$ | 5.6198 |
| **14aj** | CN | Ph-C≡C- | $-OCH_2CONHBu^t$ | 7.5850 |
| **14ak** | CN | Ph-C≡C- | $-OCH_2CN$ | 7.7447 |

# Test set for activity I; § Test set for activity II.

**Table S2** Structure–activity relationship data related to the series of 1,3-dihydro-benzo[b][1,4]diazepin-2-one derivatives described in this study (group 15).

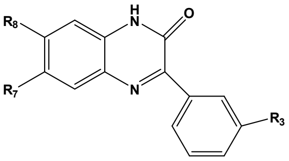

| NO | $R_3$ | $R_8$ | $R_7$ | Activity I ($pIC_{50}$) | Activity II ($pIC_{50}$) |
|---|---|---|---|---|---|
| **15a**# | 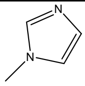 | Ph-C≡C- | H | 7.8861 | 8.0000 |
| **15b** | 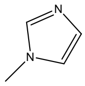 | 4-F-$C_6H_4$-C≡C- | H | 8.0458 | 8.0458 |
| **15c** | 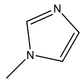 | 2-F-$C_6H_4$-C≡C- | H | 7.6990 | 7.7959 |
| **15d** | 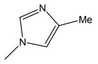 | 4-F-$C_6H_4$-C≡C- | H | 7.0088 | |
| **15e** | 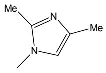 | Ph-C≡C- | H | 6.7696 | |
| **15f** | 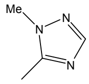 | Ph-C≡C- | H | 6.1367 | |
| **15g** | 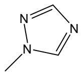 | 4-F-$C_6H_4$-C≡C- | H | 7.0088 | |
| **15h** | 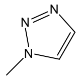 | 4-F-$C_6H_4$-C≡C- | H | 8.2218 | 7.9208 |
| **15i** | 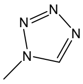 | 4-F-$C_6H_4$-C≡C- | H | 7.1427 | |
| **15j** | 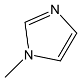 | Ph-C≡C- | -$OCH_2CH_2OMe$ | 7.6383 | 7.8239 |
| **15k** | 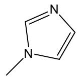 | Ph-C≡C- | -$OCH_2CH_2OH$ | 7.1938 | |
| **15l** | 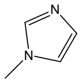 | 4-F-$C_6H_4$-C≡C- | -$OCH_2CH_2OH$ | 7.3372 | |
| **15m** | 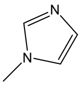 | Ph-C≡C- | -$OCH_2CN$ | 7.7447 | 7.8861 |
| **15n** | 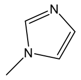 | 4-F-$C_6H_4$-C≡C- | -N(Me)$CH_2CH_2OH$ | 6.8539 | |
| **15o** | 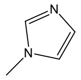 | 4-F-$C_6H_4$-C≡C- | -OH | 7.5850 | 7.9586 |
| **15p** | 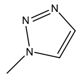 | Ph-C≡C- | -$NMe_2$ | 7.4437 | |
| **15q** | 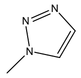 | 4-F-$C_6H_4$-C≡C- | -OH | 7.6990 | 7.7696 |
| **15r**# | 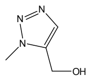 | 4-F-$C_6H_4$-C≡C- | OH | 6.4225 | |

# Test set for activity I; § Test set for activity II.

**Table S3** Structure–activity relationship data related to the series of 1,3-dihydro-benzo[b][1,4]diazepin-2-one derivatives described in this study (group 7).

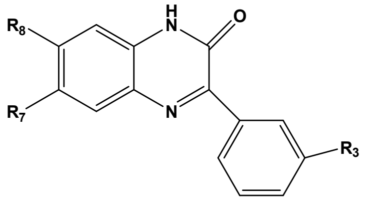


| NO | $R_3$ | $R_8$ | $R_7$ | Activity I ($pIC_{50}$) | Activity II ($pIC_{50}$) |
|---|---|---|---|---|---|
| **7a**[§] | 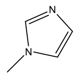 | Ph | H | 7.4089 | 7.3279 |
| **7b** | 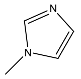 | 4-F-$C_6H_4$- | H | 7.7696 | 7.6198 |
| **7c** | 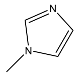 | 2-F-$C_6H_4$- | H | 7.9208 | 7.5686 |
| **7d** | 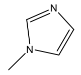 | 3-F-$C_6H_4$- | H | 7.6021 | |
| **7e** | 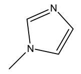 | 2,5-Di-F-$C_6H_3$ - | H | 8.1549 | 7.7696 |
| **7f** | 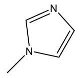 | 2-F-$C_6H_4$- | HO- | 7.6576 | |
| **7g** | 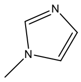 | *Cyclo*-propyl | H | 6.6778 | |
| **7h** | 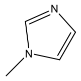 | Br | H | 7.1427 | |
| **7i**[#] | 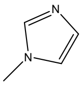 | $F_3C$- | H | 7.3665 | |
| **7j**[#] | 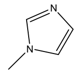 | $F_3C$- | -$NMe_2$ | 8.0458 | 7.2840 |
| **7k** | 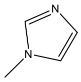 | $F_3C$- | *Iso*-but ylNH | 7.7212 | 7.1367 |
| **7l** | 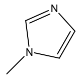 | $F_3C$- | Cl | 7.7696 | 7.8239 |
| **7m** | 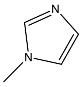 | $F_3C$- | Me | 7.9208 | 7.7212 |
| **7n** | 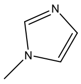 | $F_3C$- | Et | 8.3979 | 8.0000 |
| **7o** | 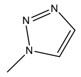 | 4-F-$C_6H_4$- | H | 7.5086 | 7.4437 |
| **7p**[#] | 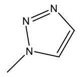 | 2-F-$C_6H_4$- | H | 7.7959 | 7.4318 |
| **7q**[#] | 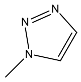 | 2,5-Di-F-$C_6H_3$ - | H | 7.7696 | 7.7447 |

**Table S3.** *Cont.*

| | | | | | |
|---|---|---|---|---|---|
| **7r** | 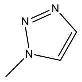 | *Iso*-propyl | H | 6.3316 | |
| **7s** | 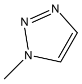 | Br | H | 6.8861 | |
| **7t**# | 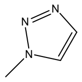 | Cl | H | 7.2218 | |
| **7u**# | 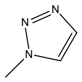 | $F_3C$- | H | 7.0362 | |
| **7v**# | 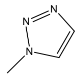 | $F_2CH$- | H | 6.2161 | |
| **7w** | 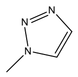 | $F_3CCH_2O$- | H | 7.1549 | |
| **7x**§ | 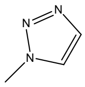 | $F_3C$- | $Me_2N$ | 7.4202 | 6.7352 |
| **7y** | 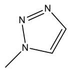 | $F_3C$- | *Iso*-butylNH | 7.3768 | 6.5719 |
| **7z** | 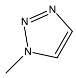 | $F_3C$- | *Iso*-butylN(Me) | 7.9586 | 7.8539 |
| **7aa** | 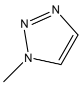 | $F_3C$- | Cl | 7.5686 | 6.9355 |
| **7ab** | 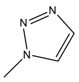 | $F_3C$- | Me | 8.0458 | 7.4089 |
| **7ac** | 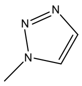 | $F_3C$- | MeO | 7.5376 | 7.1308 |
| **7ad**# | 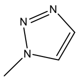 | $F_3C$- | EtO | 8.1549 | 7.4685 |

# Test set for activity I; § Test set for activity II.

**Table S4** Structure–activity relationship data related to the series of 1,3-dihydro-benzo[b][1,4]diazepin-2-one derivatives described in this study (group 8).

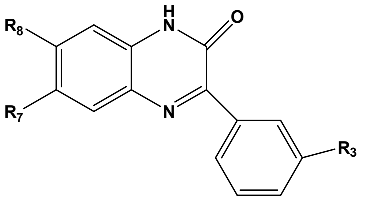

| NO | $R_3$ | $R_8$ | $R_7$ | Activity I ($pIC_{50}$) | Activity II ($pIC_{50}$) |
|---|---|---|---|---|---|
| **8a** | 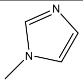 | $F_3C$- | Me | 7.9208 | 7.7212 |
| **8b** | 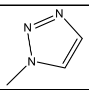 | $F_3C$- | Me | 8.0458 | 7.4089 |
| **8c** | 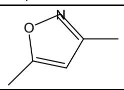 | $F_3C$- | Me | 8.3010 | 8.0458 |
| **8d** | 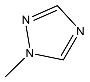 | $F_3C$- | Me | 8.0969 | 7.1249 |
| **8e** | 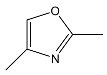 | $F_3C$- | H | 6.4559 | |
| **8f**[#] | 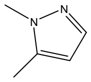 | $F_3C$- | H | 7.4089 | 7.7212 |
| **8g** | 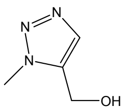 | $F_3C$- | Me | 6.9208 | |
| **8h**[§] | 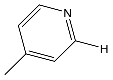 | $F_3C$- | Me | 8.3979 | 7.9586 |
| **8i**[#] | 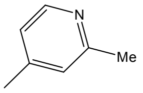 | $F_3C$- | Me | 8.3979 | 8.3979 |
| **8j** | 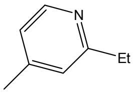 | $F_3C$- | Me | 8.0969 | 8.3010 |
| **8k**[#] [§] | 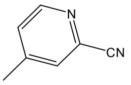 | $F_3C$- | Me | 7.9208 | 7.7447 |
| **8l** | 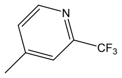 | $F_3C$- | Me | 7.1805 | |
| **8m**[§] | 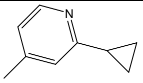 | $F_3C$- | Me | 8.0000 | 8.2218 |
| **8n** | 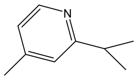 | $F_3C$- | Me | 8.3979 | 8.0000 |

**Table S4.** *Cont.*

| | | | | | |
|---|---|---|---|---|---|
| **8o** | 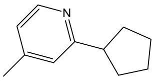 | $F_3C$- | Me | 8.0000 | 8.1549 |
| **8q** | 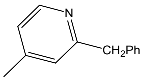 | $F_3C$- | Me | 7.7959 | 8.3010 |
| **8r** | 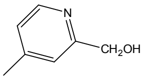 | $F_3C$- | Me | 7.8861 | 7.7447 |
| **8s** | 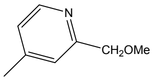 | $F_3C$- | Me | 7.8861 | 8.2218 |
| **8t** | 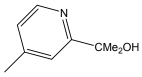 | $F_3C$- | Me | 7.3979 | 7.8539 |
| **8u** | 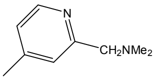 | $F_3C$- | Me | 6.5622 | |
| **8v** | 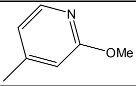 | $F_3C$- | Me | 7.8861 | 7.7959 |
| **8w** | 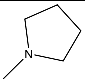 | $F_3C$- | Me | 7.2291 | |
| **8x** | 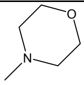 | $F_3C$- | Me | 7.5376 | |
| **8y** | 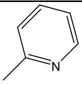 | $F_3C$- | Me | 7.5850 | 7.5229 |
| **8z** | 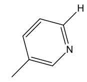 | $F_3C$- | Me | 8.6990 | 7.5229 |
| **8aa** | 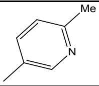 | $F_3C$- | Me | 7.8861 | 8.3010 |
| **8ab** | 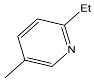 | $F_3C$- | Me | 8.0458 | 7.7447 |
| **8ac**[§] | 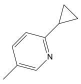 | $F_3C$- | Me | 8.2218 | 7.6990 |
| **8ad** | 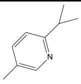 | $F_3C$- | Me | 8.0000 | 8.0000 |
| **8ae**[#] | 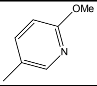 | $F_3C$- | Me | 8.3010 | 8.0000 |
| **8af** | 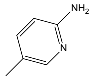 | $F_3C$- | Me | 7.8539 | 6.7959 |
| **8ag** | 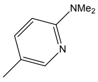 | $F_3C$- | Me | 7.0655 | |
| **8ah**[#] [§] | 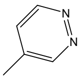 | $F_3C$- | Me | 7.7447 | 7.1427 |
| **8ai** | 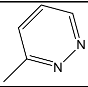 | $F_3C$- | Me | 6.8928 | |
| **8aj** | 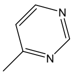 | $F_3C$- | Me | 8.2218 | 7.3010 |

**Table S4.** *Cont.*

| | | | | | |
|---|---|---|---|---|---|
| **8ak**# | 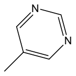 | $F_3C$- | Me | 7.6383 | 7.3188 |
| **8al** | 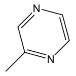 | $F_3C$- | Me | 7.6990 | 7.7959 |
| 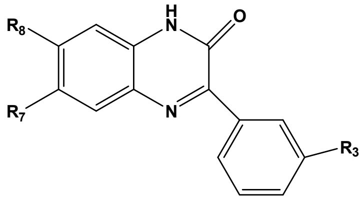 | | | | | |
| NO | $R_3$ | $R_8$ | $R_7$ | Activity I ($pIC_{50}$) | Activity II ($pIC_{50}$) |
| **8am** | 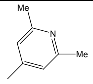 | $F_3C$- | Me | 8.6990 | 8.5229 |
| **8an** | 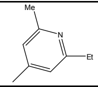 | $F_3C$- | Me | 8.6990 | 8.3979 |
| **8ao**# § | 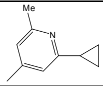 | $F_3C$- | Me | 8.6990 | 8.3979 |
| **8ap**§ | 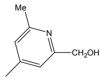 | $F_3C$- | Me | 7.8539 | 7.8239 |
| **8aq** | 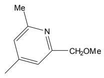 | $F_3C$- | Me | 8.0969 | 8.3010 |
| **8ar** | 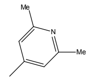 | $F_3C$- | $F_3C$- | 8.1549 | 8.1549 |
| **8as**# | 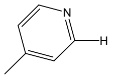 | $F_3C$- | H | 8.0000 | 7.5850 |
| **8at** | 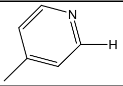 | $F_3C$- | OEt | 8.3979 | 8.0000 |
| **8au** | 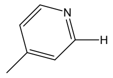 | Cl | H | 8.0969 | 8.5229 |
| **8av** | 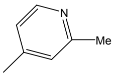 | Cl | Cl | 8.3979 | 8.6990 |
| **8aw**§ | 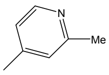 | $F_3C$ | H | 7.7447 | 7.4437 |
| **8ax** | 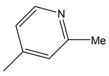 | $F_3C$ | OEt | 8.2218 | 8.0969 |

# Test set for activity I; § Test set for activity II.

**Table S5.** Observed and CoMFA/CoMSIA predicted mGluR2 inhibitory activity I ($pIC_{50}$ value).

| NO | Observed activity | CoMFA | | CoMSIA | |
|---|---|---|---|---|---|
| | | Predicted | Residual | Predicted | Residual |
| 1 | 5.19 | 5.799 | -0.609 | 6.125 | -0.935 |
| 2 | 7.47 | 7.441 | 0.029 | 7.621 | -0.151 |
| 14a | 5.18 | 5.233 | -0.053 | 5.567 | -0.387 |
| 14b | 5.55 | 5.541 | 0.009 | 5.846 | -0.296 |
| 14c | 5.82 | 6.182 | -0.362 | 6.391 | -0.571 |
| 14d | 5.54 | 5.404 | 0.136 | 5.802 | -0.262 |
| 14e | 7.59 | 7.026 | 0.564 | 6.677 | 0.913 |
| 14f | 6.02 | 6.440 | -0.420 | 6.450 | -0.430 |
| 14g | 7.10 | 6.932 | 0.168 | 6.719 | 0.381 |
| 14h | 6.57 | 6.956 | -0.386 | 6.800 | -0.230 |
| 14i | 6.81 | 7.117 | -0.307 | 6.714 | 0.096 |
| 14j | 6.45 | 6.784 | -0.334 | 6.304 | 0.146 |
| 14k | 6.44 | 6.987 | -0.547 | 6.493 | -0.053 |
| 14l | 5.98 | 6.036 | -0.056 | 6.026 | -0.046 |
| 14m | 7.47 | 7.063 | 0.407 | 7.073 | 0.397 |
| 14n | 7.12 | 6.904 | 0.216 | 6.982 | 0.138 |
| 14o | 7.19 | 7.206 | -0.016 | 7.417 | -0.227 |
| 14p | 7.17 | 6.869 | 0.301 | 7.144 | 0.026 |
| 14q | 7.47 | 7.285 | 0.185 | 7.408 | 0.062 |
| 14r | 7.52 | 7.295 | 0.225 | 7.433 | 0.087 |
| 14s | 6.32 | 6.262 | 0.058 | 6.381 | -0.061 |
| 14t | 7.49 | 7.485 | 0.005 | 7.668 | -0.178 |
| 14u | 6.52 | 6.626 | -0.106 | 6.753 | -0.233 |
| 14v | 6.06 | 6.127 | -0.067 | 6.081 | -0.021 |
| 14w | 4.59 | 5.086 | -0.496 | 4.204 | 0.386 |
| 14x | 6.40 | 5.847 | 0.553 | 6.090 | 0.310 |
| 14y | 7.22 | 6.883 | 0.337 | 7.207 | 0.013 |
| 14z | 6.42 | 6.474 | -0.054 | 6.814 | -0.394 |
| 14aa | 6.56 | 6.529 | 0.031 | 6.866 | -0.306 |
| 14ab | 7.40 | 7.088 | 0.312 | 7.626 | -0.226 |
| 14ac | 6.35 | 6.587 | -0.237 | 7.064 | -0.714 |
| 14ad | 6.80 | 6.592 | 0.208 | 7.136 | -0.336 |
| 14ae | 7.09 | 6.640 | 0.450 | 7.119 | -0.029 |
| 14af | 7.04 | 7.035 | 0.005 | 7.229 | -0.189 |
| 14ag | 7.55 | 7.395 | 0.155 | 7.738 | -0.188 |
| 14ah | 6.22 | 6.233 | -0.013 | 6.020 | 0.200 |
| 14ai | 5.62 | 5.843 | -0.223 | 5.660 | -0.040 |
| 14aj | 7.59 | 7.515 | 0.075 | 7.620 | -0.030 |
| 14ak | 7.74 | 8.063 | -0.323 | 7.918 | -0.178 |
| 15a | 7.89 | 7.484 | 0.406 | 7.758 | 0.132 |
| 15b | 8.05 | 7.959 | 0.091 | 7.858 | 0.192 |
| 15c | 7.70 | 7.359 | 0.341 | 7.577 | 0.123 |
| 15d | 7.01 | 7.032 | -0.022 | 7.009 | 0.001 |
| 15e | 6.77 | 7.105 | -0.335 | 6.672 | 0.098 |
| 15f | 6.14 | 6.158 | -0.018 | 6.069 | 0.071 |

**Table S5.** *Cont.*

| | | | | | |
|---|---|---|---|---|---|
| 15g | 7.01 | 7.640 | -0.630 | 7.370 | -0.360 |
| 15h | 8.22 | 8.130 | 0.090 | 7.524 | 0.696 |
| 15i | 7.14 | 7.322 | -0.182 | 7.046 | 0.094 |
| 15j | 7.64 | 7.710 | -0.070 | 7.728 | -0.088 |
| 15k | 7.19 | 6.941 | 0.249 | 7.336 | -0.146 |
| 15l | 7.34 | 7.547 | -0.207 | 7.294 | 0.046 |
| 15m | 7.74 | 7.719 | 0.021 | 7.754 | -0.014 |
| 15n | 6.85 | 6.704 | 0.146 | 6.616 | 0.234 |
| 15o | 7.59 | 7.470 | 0.120 | 7.421 | 0.169 |
| 15p | 7.44 | 7.591 | -0.151 | 7.469 | -0.029 |
| 15q | 7.70 | 7.379 | 0.321 | 7.857 | -0.157 |
| 15r | 6.42 | 6.258 | 0.162 | 6.453 | -0.033 |
| 7a | 7.41 | 7.466 | -0.056 | 7.665 | -0.255 |
| 7b | 7.77 | 7.651 | 0.119 | 7.737 | 0.033 |
| 7c | 7.92 | 7.793 | 0.127 | 7.777 | 0.143 |
| 7d | 7.60 | 7.582 | 0.018 | 7.660 | -0.060 |
| 7e | 8.15 | 7.850 | 0.300 | 7.745 | 0.405 |
| 7f | 7.66 | 7.427 | 0.233 | 7.727 | -0.067 |
| 7g | 6.68 | 6.832 | -0.152 | 6.998 | -0.318 |
| 7h | 7.14 | 7.270 | -0.130 | 6.959 | 0.181 |
| 7i | 7.37 | 7.462 | -0.092 | 7.877 | -0.507 |
| 7j | 8.05 | 7.830 | 0.220 | 7.989 | 0.061 |
| 7k | 7.72 | 7.226 | 0.494 | 7.632 | 0.088 |
| 7l | 7.77 | 7.829 | -0.059 | 8.049 | -0.279 |
| 7m | 7.92 | 8.101 | -0.181 | 8.118 | -0.198 |
| 7n | 8.40 | 8.148 | 0.252 | 8.181 | 0.219 |
| 7o | 7.51 | 7.241 | 0.269 | 7.293 | 0.217 |
| 7p | 7.80 | 7.364 | 0.436 | 7.311 | 0.489 |
| 7q | 7.77 | 7.410 | 0.360 | 7.280 | 0.490 |
| 7r | 6.33 | 6.752 | -0.422 | 6.804 | -0.474 |
| 7s | 6.89 | 6.373 | 0.517 | 6.288 | 0.602 |
| 7t | 7.22 | 6.739 | 0.481 | 7.188 | 0.032 |
| 7u | 7.04 | 6.978 | 0.062 | 7.397 | -0.357 |
| 7v | 6.22 | 6.283 | -0.063 | 6.613 | -0.393 |
| 7w | 7.15 | 7.266 | -0.116 | 7.319 | -0.169 |
| 7x | 7.42 | 7.560 | -0.140 | 7.642 | -0.222 |
| 7y | 7.38 | 7.583 | -0.203 | 7.502 | -0.122 |
| 7z | 7.96 | 8.008 | -0.048 | 7.931 | 0.029 |
| 7aa | 7.57 | 7.683 | -0.113 | 7.707 | -0.137 |
| 7ab | 8.05 | 7.953 | 0.097 | 8.024 | 0.026 |
| 7ac | 7.54 | 7.744 | -0.204 | 7.650 | -0.110 |
| 7ad | 8.15 | 8.256 | -0.106 | 8.086 | 0.064 |
| 8a | 7.92 | 8.144 | -0.224 | 7.982 | -0.062 |
| 8b | 8.05 | 7.953 | 0.097 | 8.024 | 0.026 |
| 8c | 8.30 | 8.113 | 0.187 | 7.980 | 0.320 |
| 8d | 8.10 | 7.414 | 0.686 | 7.720 | 0.380 |
| 8e | 6.46 | 6.872 | -0.412 | 6.729 | -0.269 |
| 8f | 7.41 | 7.346 | 0.064 | 7.463 | -0.053 |

**Table S5.** *Cont.*

| | | | | | |
|---|---|---|---|---|---|
| 8g | 6.92 | 6.626 | 0.294 | 6.618 | 0.302 |
| 8h | 8.40 | 8.517 | -0.117 | 8.277 | 0.123 |
| 8i | 8.40 | 8.374 | 0.026 | 8.190 | 0.210 |
| 8j | 8.10 | 8.404 | -0.304 | 8.167 | -0.067 |
| 8k | 7.92 | 7.670 | 0.250 | 7.106 | 0.814 |
| 8l | 7.18 | 7.583 | -0.403 | 7.240 | -0.060 |
| 8m | 8.00 | 7.341 | 0.659 | 7.723 | 0.277 |
| 8n | 8.40 | 7.994 | 0.406 | 7.983 | 0.417 |
| 8o | 8.00 | 7.629 | 0.371 | 7.578 | 0.422 |
| 8p | 6.93 | 7.297 | -0.367 | 7.222 | -0.292 |
| 8q | 7.80 | 7.881 | -0.081 | 7.934 | -0.134 |
| 8r | 7.89 | 8.193 | -0.303 | 7.881 | 0.009 |
| 8s | 7.89 | 8.249 | -0.359 | 7.675 | 0.215 |
| 8t | 7.40 | 7.436 | -0.036 | 7.137 | 0.263 |
| 8u | 6.56 | 7.181 | -0.621 | 7.223 | -0.663 |
| 8v | 7.89 | 7.929 | -0.039 | 7.827 | 0.063 |
| 8w | 7.23 | 7.121 | 0.109 | 7.320 | -0.090 |
| 8x | 7.54 | 7.404 | 0.136 | 7.443 | 0.097 |
| 8y | 7.59 | 7.926 | -0.336 | 7.600 | -0.010 |
| 8z | 8.70 | 8.065 | 0.635 | 8.099 | 0.601 |
| 8aa | 7.89 | 7.728 | 0.162 | 7.896 | -0.006 |
| 8ab | 8.05 | 8.063 | -0.013 | 8.068 | -0.018 |
| 8ac | 8.22 | 7.913 | 0.307 | 8.115 | 0.105 |
| 8ad | 8.00 | 7.912 | 0.088 | 7.971 | 0.029 |
| 8ae | 8.30 | 7.992 | 0.308 | 7.997 | 0.303 |
| 8af | 7.85 | 7.972 | -0.122 | 7.856 | -0.006 |
| 8ag | 7.07 | 7.606 | -0.536 | 7.901 | -0.831 |
| 8ah | 7.74 | 7.843 | -0.103 | 7.850 | -0.110 |
| 8ai | 6.89 | 7.389 | -0.499 | 7.445 | -0.555 |
| 8aj | 8.22 | 7.808 | 0.412 | 7.836 | 0.384 |
| 8ak | 7.64 | 7.618 | 0.022 | 7.788 | -0.148 |
| 8al | 7.70 | 7.592 | 0.108 | 7.363 | 0.337 |
| 8am | 8.70 | 8.666 | 0.034 | 8.527 | 0.173 |
| 8an | 8.70 | 8.706 | -0.006 | 8.466 | 0.234 |
| 8ao | 8.70 | 8.313 | 0.387 | 8.416 | 0.284 |
| 8ap | 7.85 | 8.133 | -0.283 | 7.957 | -0.107 |
| 8aq | 8.10 | 7.756 | 0.344 | 7.875 | 0.225 |
| 8ar | 8.15 | 8.429 | -0.279 | 8.595 | -0.445 |
| 8as | 8.00 | 7.901 | 0.099 | 7.929 | 0.071 |
| 8at | 8.40 | 8.448 | -0.048 | 8.496 | -0.096 |
| 8au | 8.10 | 7.686 | 0.414 | 7.808 | 0.292 |
| 8av | 8.40 | 8.190 | 0.210 | 8.298 | 0.102 |
| 8aw | 7.74 | 7.763 | -0.270 | 8.007 | -0.267 |
| 8ax | 8.22 | 8.338 | 0.420 | 8.462 | -0.242 |

**Table S6**. Observed and CoMFA/CoMSIA predicted mGluR2 inhibitory activity II ($pIC_{50}$ value).

| NO | Observed activity | CoMFA | | CoMSIA | |
|---|---|---|---|---|---|
| | | Predicted | Residual | Predicted | Residual |
| 2 | 7.77 | 7.935 | -0.165 | 6.970 | -0.400 |
| 15a | 8.00 | 7.939 | 0.061 | 7.581 | -0.861 |
| 15b | 8.05 | 7.900 | 0.150 | 7.712 | -0.912 |
| 15c | 7.80 | 8.037 | -0.237 | 7.248 | -0.308 |
| 15h | 7.92 | 8.000 | -0.080 | 7.118 | 0.002 |
| 15j | 7.82 | 8.006 | -0.186 | 7.080 | 0.050 |
| 15m | 7.89 | 7.905 | -0.015 | 7.338 | -0.198 |
| 15o | 7.96 | 7.945 | 0.015 | 7.496 | -0.216 |
| 15q | 7.77 | 7.564 | 0.206 | 7.660 | -0.360 |
| 7a | 7.33 | 7.606 | -0.276 | 7.564 | -0.244 |
| 7b | 7.62 | 7.669 | -0.049 | 7.129 | 0.341 |
| 7c | 7.57 | 7.640 | -0.070 | 8.018 | -0.498 |
| 7e | 7.77 | 7.695 | 0.075 | 7.777 | -0.207 |
| 7j | 7.28 | 7.410 | -0.130 | 7.707 | -0.117 |
| 7k | 7.14 | 7.004 | 0.136 | 7.797 | -0.177 |
| 7l | 7.82 | 7.543 | 0.277 | 7.644 | 0.076 |
| 7m | 7.72 | 7.562 | 0.158 | 7.656 | 0.064 |
| 7n | 8.00 | 7.676 | 0.324 | 7.404 | 0.336 |
| 7o | 7.44 | 7.604 | -0.164 | 7.976 | -0.236 |
| 7p | 7.43 | 7.552 | -0.122 | 7.919 | -0.179 |
| 7q | 7.74 | 7.630 | 0.110 | 7.806 | -0.036 |
| 7x | 6.74 | 7.312 | -0.572 | 7.398 | 0.372 |
| 7y | 6.57 | 6.446 | 0.124 | 8.175 | -0.375 |
| 7z | 7.85 | 7.822 | 0.028 | 7.599 | 0.201 |
| 7aa | 6.94 | 7.157 | -0.217 | 7.230 | 0.180 |
| 7ab | 7.41 | 7.423 | -0.013 | 7.225 | 0.185 |
| 7ac | 7.13 | 7.221 | -0.091 | 7.376 | 0.054 |
| 7ad | 7.47 | 7.369 | 0.101 | 7.392 | 0.048 |
| 8a | 7.72 | 7.576 | 0.144 | 8.001 | -0.201 |
| 8aa | 8.30 | 7.746 | 0.554 | 7.716 | 0.104 |
| 8ab | 7.74 | 7.866 | -0.126 | 7.971 | -0.151 |
| 8ac | 7.70 | 7.842 | -0.142 | 8.007 | -0.157 |
| 8ad | 8.00 | 7.976 | 0.024 | 7.417 | 0.433 |
| 8ae | 8.00 | 7.893 | 0.107 | 7.792 | 0.098 |
| 8af | 6.80 | 7.507 | -0.707 | 7.768 | 0.152 |
| 8ah | 7.14 | 7.430 | -0.290 | 8.026 | -0.066 |
| 8aj | 7.30 | 7.535 | -0.235 | 8.334 | -0.334 |
| 8ak | 7.32 | 7.366 | -0.046 | 7.752 | 0.248 |
| 8al | 7.80 | 7.453 | 0.347 | 7.890 | 0.110 |
| 8am | 8.52 | 8.353 | 0.167 | 7.989 | 0.011 |
| 8an | 8.40 | 8.067 | 0.333 | 7.909 | 0.091 |
| 8ao | 8.40 | 8.442 | -0.042 | 8.007 | -0.007 |
| 8ap | 7.82 | 8.056 | -0.236 | 8.089 | -0.039 |
| 8aq | 8.30 | 8.121 | 0.179 | 7.879 | 0.171 |
| 8ar | 8.15 | 7.978 | 0.172 | 8.051 | 0.099 |
| 8as | 7.59 | 7.582 | 0.008 | 8.440 | -0.290 |

**Table S6.** *Cont.*

| | | | | | |
|---|---|---|---|---|---|
| 8at | 8.00 | 7.962 | 0.038 | 8.057 | 0.163 |
| 8au | 8.52 | 8.184 | 0.336 | 8.174 | 0.126 |
| 8av | 8.70 | 8.549 | 0.151 | 8.227 | 0.073 |
| 8aw | 7.44 | 7.679 | -0.239 | 7.853 | 0.447 |
| 8ax | 8.10 | 8.107 | -0.007 | 8.431 | -0.131 |
| 8b | 7.41 | 7.418 | -0.008 | 8.121 | 0.279 |
| 8c | 8.05 | 8.207 | -0.157 | 8.082 | 0.318 |
| 8d | 7.12 | 7.210 | -0.090 | 7.906 | 0.614 |
| 8f | 6.72 | 7.446 | -0.726 | 8.034 | 0.486 |
| 8h | 7.96 | 7.969 | -0.009 | 8.262 | 0.258 |
| 8i | 8.40 | 8.123 | 0.277 | 8.215 | 0.485 |
| 8j | 8.30 | 8.269 | 0.031 | 7.089 | -0.349 |
| 8k | 7.74 | 7.962 | -0.222 | 7.578 | -0.438 |
| 8m | 8.22 | 8.240 | -0.020 | 7.734 | -0.404 |
| 8n | 8.00 | 8.371 | -0.371 | 7.807 | -0.367 |
| 8o | 8.15 | 8.504 | -0.354 | 7.945 | -0.245 |
| 8q | 8.30 | 8.547 | -0.247 | 7.920 | -0.180 |
| 8r | 7.74 | 7.902 | -0.162 | 7.891 | -0.121 |
| 8s | 8.22 | 8.021 | 0.199 | 8.117 | -0.297 |
| 8t | 7.85 | 7.938 | -0.088 | 8.009 | -0.049 |
| 8v | 7.80 | 8.143 | -0.343 | 8.023 | 0.077 |
| 8y | 7.52 | 7.886 | -0.366 | 8.271 | -0.051 |
| 8z | 8.52 | 7.974 | 0.546 | 8.364 | 0.036 |

**Figure S1.** CoMSIA StDev*Coeff contour maps for activityII.(**A**) Electrostatic contour map (red/blue) in combination with compound **8av**. Red contours indicate regions where negative charges increase activity; blue contours indicate regions where positive charges increase activity; (**b**) Hydrophobic contour map (yellow/white) in combination with compound **8av**. Yellow contours indicate regions where hydrophobic substituents enhance activity; white contours indicate regions where hydrophilic substituents enhance activity II.

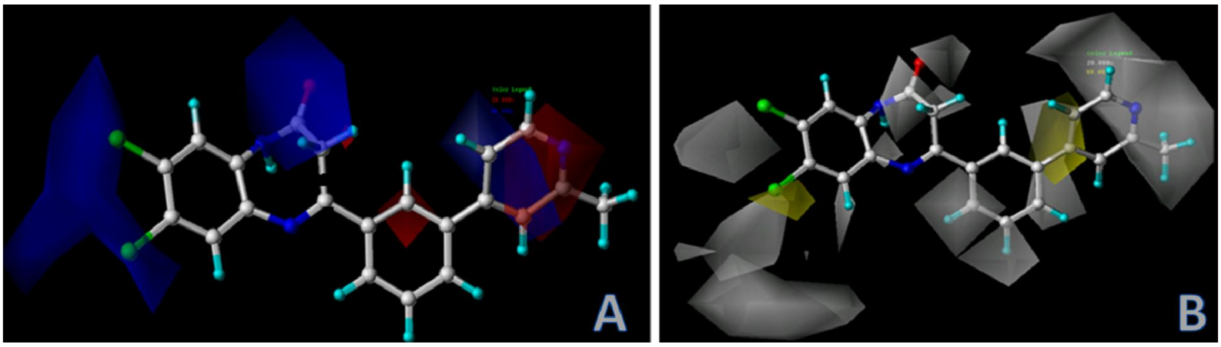

**Figure S2.** The ligand-based correlation plots of the CoMSIA predicted versus the actual $pIC_{50}$ values using the training (filled black square) and the test (filled blue circle) sets for activity II.

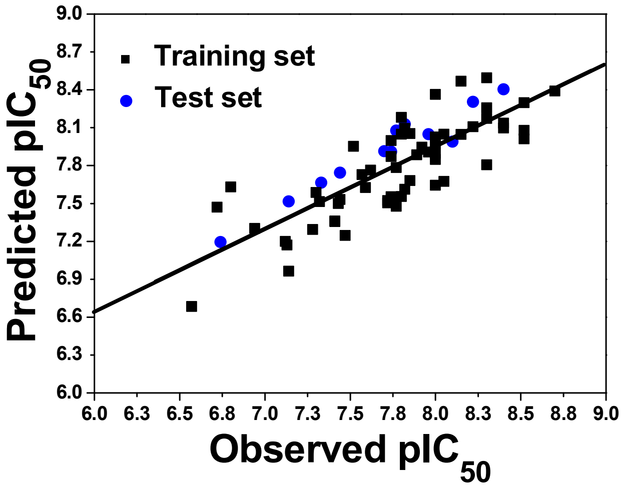


**Figure S3.** Homology modeling result sheet for mGluR2.

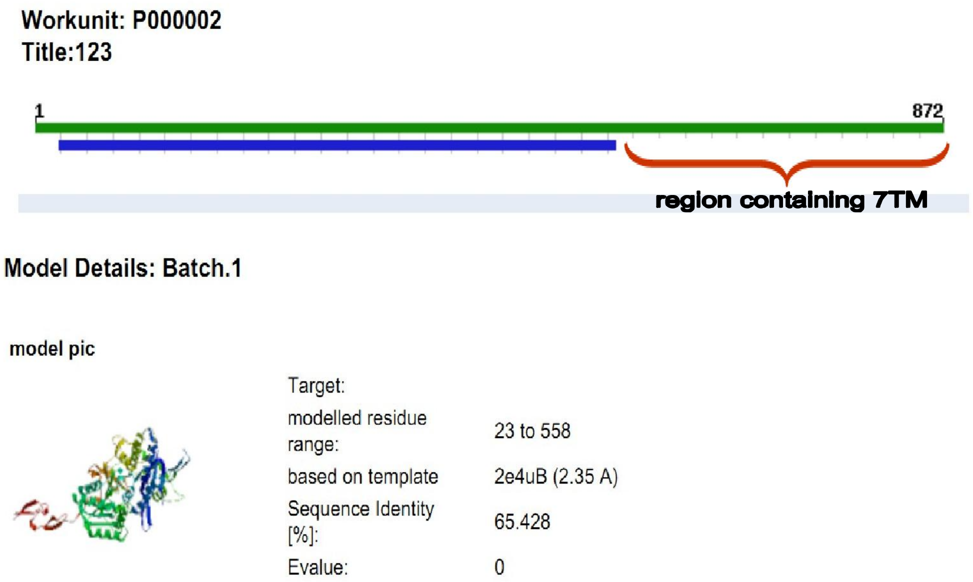


© 2011 by the authors; licensee MDPI, Basel, Switzerland. This article is an open access article distributed under the terms and conditions of the Creative Commons Attribution license (http://creativecommons.org/licenses/by/3.0/).
